# The varicella-zoster virus ORF16 protein promotes both the nuclear transport and the protein abundance of the viral DNA polymerase subunit ORF28

**DOI:** 10.1016/j.virusres.2024.199379

**Published:** 2024-04-26

**Authors:** Huang-Shen Lin, Cheng-Han Li, Lee-Wen Chen, Shie-Shan Wang, Li-Yu Chen, Chien-Hui Hung, Chun-Liang Lin, Pey-Jium Chang

**Affiliations:** a Department of Internal Medicine, Division of Infectious Diseases, Chang Gung Memorial Hospital, Chiayi 61363, Taiwan; b Graduate Institute of Clinical Medical Sciences, College of Medicine, Chang Gung University, Taoyuan 33302, Taiwan; c Department of Respiratory Care, Chang Gung University of Science and Technology, Chiayi 61363, Taiwan; d Department of Pediatric Surgery, Chang Gung Memorial Hospital, Chiayi 61363, Taiwan; e School of Medicine, Chang Gung University, Taoyuan 33302, Taiwan; f Department of Nephrology, Chang Gung Memorial Hospital, Chiayi 61363, Taiwan

**Keywords:** VZV, ORF16, ORF28, Nuclear transport, Protein stability, Hsp90

## Abstract

Although all herpesviruses utilize a highly conserved replication machinery to amplify their viral genomes, different members may have unique strategies to modulate the assembly of their replication components. Herein, we characterize the subcellular localization of seven essential replication proteins of varicella-zoster virus (VZV) and show that several viral replication enzymes such as the DNA polymerase subunit ORF28, when expressed alone, are localized in the cytoplasm. The nuclear import of ORF28 can be mediated by the viral DNA polymerase processivity factor ORF16. Besides, ORF16 could markedly enhance the protein abundance of ORF28. Noteworthily, an ORF16 mutant that is defective in nuclear transport still retained the ability to enhance ORF28 abundance. The low abundance of ORF28 in transfected cells was due to its rapid degradation mediated by the ubiquitin-proteasome system. We additionally reveal that radicicol, an inhibitor of the chaperone Hsp90, could disrupt the interaction between ORF16 and ORF28, thereby affecting the nuclear entry and protein abundance of ORF28. Collectively, our findings imply that the cytoplasmic retention and rapid degradation of ORF28 may be a key regulatory mechanism for VZV to prevent untimely viral DNA replication, and suggest that Hsp90 is required for the interaction between ORF16 and ORF28.

## Highlights

• Several VZV-encoded replication enzymes *per se* are localized in the cytoplasm.
• The nuclear import of VZV ORF28 can be mediated by ORF16.
• ORF16 can enhance the protein abundance of ORF28.
• The low abundance of ORF28 in cells is due to rapid degradation by the proteasome.
• Radicicol, a Hsp90 inhibitor, can disrupt the interaction between ORF16 and ORF28.

## 1 Introduction

Herpesviruses are double-stranded DNA viruses critically associated with a wide variety of human diseases (Sehrawat et al., 2018). Since herpesviruses encode their own DNA replication proteins to amplify viral genomes in the cell nucleus, they are considered as excellent model systems for studying the mechanism and regulation of eukaryotic DNA replication (Packard and Dembowski, 2021; Weller and Coen, 2012). Moreover, due to the essential roles in viral propagation, the viral replication enzymes often serve as antiviral drug targets (James and Prichard, 2014; Yajima et al., 2017; Zarrouk et al., 2017). Therefore, understanding subunit interactions of the viral DNA replication complex in detail may provide a strong foundation for the development of new strategies or novel compounds against viral infection.

Studies on the lytic replication of herpes simplex virus type 1 (HSV-1), the prototype of the *Alphaherpesvirus* subfamily, have shown that HSV-1 encodes seven proteins essential for viral DNA synthesis (Packard and Dembowski, 2021; Weller and Coen, 2012). These viral proteins include the origin-binding protein (UL9) and six conserved core replication protein, namely, UL5 (helicase, HEL), UL8 (primase-associated factor, PAF), UL52 (primase, PRI), UL30 (DNA polymerase, POL), UL42 (polymerase processivity factor, PPF), and UL29 (or ICP8; single-stranded DNA-binding protein, SSB) (Table 1). According to the current model of the herpesviral DNA replication, UL30 and UL42 can form a heterodimeric replisome subcomplex, whereas UL5, UL8 and UL52 form a heterotrimeric helicase-primase subcomplex (Alvisi et al., 2013; Weller and Coen, 2012). Similarly, in human cytomegalovirus (HCMV), a member of the *Betaherpesvirus* subfamily, the DNA polymerase subunit (UL54) and the processivity factor (UL44) assemble as a replisome subcomplex (Alvisi et al., 2005, 2006), and three replication proteins including UL105 (helicase), UL70 (primase) and UL102 (primase-associated factor) assemble as a helicase-primase subcomplex (McMahon and Anders, 2002). Furthermore, two other well-known members of the *Gammaherpesvirus* subfamily, Epstein-Barr virus (EBV) and Kaposi's sarcoma-associated herpesvirus (KSHV), also encode a conserved set of DNA replication proteins (Table 1). In the case of EBV, the viral proteins essential for lytic DNA replication are the origin-binding protein (BZLF1 or Zta), the replisome subcomplex (BALF5 and BMRF1), the helicase-primase subcomplex (BBLF4, BSLF1 and BBLF2/3), and the single-stranded DNA-binding protein (BALF2) (Fixman et al., 1992, 1995; Hammerschmidt and Sugden, 2013). For the lytic DNA replication of KSHV, there are eight essential replication proteins, including two origin-binding proteins (ORF50 and K8), the replisome subcomplex (consisting of ORF9 and ORF59), the helicase-primase subcomplex (consisting of ORF44, ORF56 and ORF40/41), as well as the single-stranded DNA-binding protein (ORF6) (Aneja and Yuan, 2017; Wang et al., 2006; Wu et al., 2001).

**Table 1:** Herpesvirus genes required for lytic DNA replication.

VZV gene	Function	HSV-1 homolog	HCMV homolog	EBV homolog	KSHV homolog
ORF6	Primase (PRI)	UL52	UL70	BSLF1	ORF56
ORF16	Polymerase processivity factor (PPF)	UL42	UL44	BMRF1	ORF59
ORF28	DNA polymerase (POL)	UL30	UL54	BALF5	ORF9
ORF29	Single-stranded DNA binding (SSB)	UL29	UL57	BALF2	ORF6
ORF51	Origin-binding protein (OBP)	UL9	UL84 & IE2	BZLF1	ORF-K8
ORF52	Primase-associated factor (PAF)	UL8	UL102	BBLF2/3	ORF40/41
ORF55	Helicase (HEL)	UL5	UL105	BBLF4	ORF44

Although several studies have shown that all known herpesviruses appear to use very similar replication machineries to amplify their viral genomes, different herpesviruses may have developed distinct strategies to coordinately regulate the assembly of their replication components or subcomplexes in the nucleus. For example, studies on the replisome subcomplex in HSV-1 and HCMV displayed that both their POL enzymes (UL30 and UL54, respectively) and PPF subunits (UL42 and UL44, respectively) possess nuclear localization signals (NLSs), thereby allowing them to localize into the cell nucleus independently of other viral proteins (Alvisi et al., 2013, 2007, 2006; Loregian et al., 2000). However, in the case of EBV and KSHV, the POL enzymes (BALF5 and ORF9, respectively) do not contain a functional NLS and the nuclear import of the POL enzymes requires the interaction with their respective PPFs (BMRF1 and ORF59) that contain a functional NLS (Chen et al., 2023, 2005; Kawashima et al., 2013). Similarly, studies on veterinary herpesviruses such as pseudorabies virus (PRV) and bovine herpesvirus 1 (BoHV-1) also showed that their POL enzymes are imported into the nucleus only in the presence of their PPFs (Raza et al., 2020; Wang et al., 2016).

Varicella-zoster virus (VZV), a member of the *Alphaherpesvirus* subfamily, can cause varicella (chickenpox) during primary infection and herpes zoster (shingles) upon reactivation of the virus from latency in sensory ganglia (Gershon et al., 2015). According to the estimation of the world health organization (WHO) in 2014, there were approximately 4.2 million of varicella cases with severe complications, leading to hospitalization and 4200 deaths per year in the world (Varela et al., 2019). For herpes zoster, the incidence and severity of this disease increase with age, with a sharp increase after 50 years of age. The estimated incidence of herpes zoster in the population ≥50 years of age ranged from 5.23 to 10.9 cases per 1000 person-years (Johnson et al., 2015; van Oorschot et al., 2021). Similar to other herpesviruses, VZV encodes six conserved core replication proteins and an origin-binding protein (ORF51) responsible for viral lytic DNA replication (Khalil et al., 2015; Lenac Rovis et al., 2013). These six core replication proteins include ORF6 (primase), ORF16 (polymerase processivity factor), ORF28 (DNA polymerase), ORF29 (single-stranded DNA binding protein), ORF52 (primase-associated factor), and ORF55 (helicase) (Table 1). In comparison with other human herpesviruses, little is known about the subcellular localization of individual VZV replication protein and the physical interaction and assembly of these VZV replication proteins. To date, only the VZV ORF29 protein has been previously shown to localize to the nucleus in the absence of other viral proteins, and a noncanonical NLS was found between amino acids 9 and 154 of the protein (Stallings and Silverstein, 2005).

Since the nuclear import of herpesviral replication proteins or subcomplexes is a prerequisite for viral DNA amplification, we here aimed to investigate the subcellular localization of each VZV replication protein and attempted to determine the potential regulation of assembly of VZV replication proteins in the cell nucleus.

## 2 Materials and methods

### 2.1 Cell cultures, DNA transfections, and chemical reagents

The human embryonic kidney cell line 293T (Graham et al., 1977) was grown in high-glucose DMEM supplemented with 10 % fetal bovine serum (FBS). DNA transfection in 293T cells was performed using Lipofectamine 2000 reagent (no. 11,668,019; Thermo Fisher Scientific) (Dalby et al., 2004) according to the manufacturer's protocols. Generally, cells were harvested 24 h after transfection to analyze the expression and the protein localization of transfected genes. MG132 (no. C2211; Sigma-Aldrich), chloroquine diphosphate (no. ab142116; Abcam), and radicicol (no. R2146; Sigma-Aldrich) were purchased commercially.

### 2.2 Plasmid construction

The expression plasmids encoding VZV replication proteins were constructed by inserting individual coding regions from the genome of the VZV vOka vaccine strain (GenBank accession number: AB097932.1) into pFLAG-CMV-2 (no. E7398; Sigma-Aldrich) or into pEGFP-C2 (no. 6083–1; Clontech). The resultant plasmids were named pCMV-F-ORF6, pCMV-F-ORF16, pCMV-F-ORF28, pCMV-F-ORF29, pCMV-F-ORF51, pCMV-F-ORF52 and pCMV-F-ORF55, as well as pCMV-GFP-ORF6, pCMV-GFP-ORF16, pCMV-GFP-ORF28, pCMV-GFP-ORF29, pCMV-GFP-ORF51, pCMV-GFP-ORF52 and pCMV-GFP-ORF55. These constructed plasmids express full-length VZV replication proteins with an N-terminal FLAG tag or GFP tag. To generate the plasmids encoding deletion mutants of GFP-ORF16, the corresponding DNA fragments were amplified by PCR and then inserted into pEGFP-C2. The NLS-mutated construct of GFP-ORF16 was generated in pCMV-GFP-ORF16 using QuikChange site-directed mutagenesis kit (no. 200,524; Agilent Technologies). To construct the plasmids expressing F-(NLS)-ORF28 or F-ORF28-(NLS), a small DNA fragment encoding a functional NLS motif (amino acid residues: PKKKRKV) derived from the SV40 T antigen (Lanford and Butel, 1984) was inserted upstream or downstream of the ORF28 coding region in pCMV-F-ORF28.

### 2.3 Western blot analysis

Western blotting was performed as mentioned previously (Chen et al., 2020). In brief, cell samples were harvested and lysed in either the electrophoresis sample buffer (containing 62.5 mM Tris–HCl [pH 6.8], 2 % sodium dodecyl sulfate [SDS], 10 % glycerol, and 5 % 2-mercaptoethanol) or the coimmunoprecipitation assay buffer (containing 10 mM Tris–HCl [pH 7.5], 150 mM NaCl, 0.5 mM EDTA, 1 % Deoxycholate, and 1 % Triton X-100). Protein lysates were loaded and separated in 8–15 % SDS-polyacrylamide gel. The separated proteins in the gel were subsequently transferred onto a polyvinylidene difluoride (PVDF) membrane (no. IEBH85R; Millipore) and were probed with specific antibodies. Antibodies to FLAG (no. A8592; Sigma-Aldrich), GFP (no. G1544, Sigma-Aldrich), p27 (no. sc-1641; Santa Cruz), LC3B (no. GTX127375; GeneTex), and actin (no. sc-47,778; Santa Cruz), as well as the secondary antibodies including Goat anti-Mouse IgG (*H* + *L*) antibody conjugated with horseradish peroxidase (no. AP124P; Sigma-Aldrich) and Goat anti-Rabbit IgG (*H* + *L*) antibody conjugated with horseradish peroxidase (no. 5220–0336, SeraCare) were obtained commercially. The Western Lightning chemiluminescence reagent (no. NEL105001EA; PerkinElmer) was used for detecting the antigen-antibody complex on membranes. The signals of the antigen-antibody complex on blots were generated by exposure to X-ray film or using e-BLOT Touch imager (Biofargo). To quantify the relative intensity of target bands in Western blots, densitometry analysis of protein bands was carried out using Quantity One software (Bio-Rad), and β-actin was used as a normalizing protein.

### 2.4 Confocal fluorescence microscopic analysis

Detailed procedures for immunofluorescence staining and confocal microscopic analysis were described previously (Chen et al., 2023; Yen et al., 2021). Briefly, 293T cells were seeded at a density of 1.5 × 10^5^ cells per well in 6-well tissue culture plates and were transiently transfected with expression plasmids for 24 h. Cells were fixed with 4 % paraformaldehyde in phosphate-buffered saline (PBS) at room temperature for 10 min, followed by three PBS washes (each 5 min). For immunofluorescence staining, the fixed cells were permeabilized with 0.1 % Triton X-100 in PBS for 8 min at room temperature. Following a PBS rinse, the cells were incubated with blocking solution (CAS-Block; Invitrogen) at room temperature for 30 min and then treated with specific primary antibodies for 1 h. Specific primary antibodies against the FLAG tag (no. F1804 or F7425; Sigma-Aldrich) and Hsp90 (no. SC13119, Santa Cruz) were used in the study. After three additional washes, suitable secondary antibodies were applied, including goat anti-mouse IgG antibody/Alexa Fluor 594 (no. A-11,005; Thermo Fisher Scientific), goat anti-mouse IgG2a/Alexa Fluor 647 (no. A21241; Invitrogen), and goat anti-rabbit IgG/Alexa Fluor 594 (no. A11012; Invitrogen). Staining with 4′,6-diamidino-2-phenylindole (DAPI) was performed at room temperature for 15 min. Subsequently, the slides were mounted in Aqua Mounter (no. BSB0092; BioSB) and analyzed using a Leica confocal laser scanning system (TCS-SP5II). The excitation wavelengths (Ex) and collected wavelengths (Em) of fluorophores used in the study are as follows: Alexa Fluor 647 (Ex: 633 nm; Em: 650–720 nm), Alexa Fluor 594 (Ex: 561 nm; Em: 570–620 nm), GFP (Ex: 488 nm; Em: 500–560 nm), and DAPI (Ex: 405 nm; Em: 420–475 nm). Image analysis was conducted using LAS AF Lite software (Leica SP8 FALCON confocal microscope).

### 2.5 Quantification of nuclear localization of proteins

Quantification of nuclear accumulation of proteins of interest was performed as described previously (Alvisi et al., 2018). Briefly, both the nuclear fluorescence (Fn) and cytoplasmic fluorescence (Fc) from single cell were measured using Image J software (version 1.52 p, National Institutes of Health, Bethesda, MD). After the subtraction of background fluorescence, the results were presented as the ratio of nuclear to cytoplasmic fluorescence (Fn/c).

### 2.6 Coimmunoprecipitation

Coimmunoprecipitation assays were performed as described previously (Chang et al., 2016). In brief, after transfection of the expression plasmids into 293T cells for 24 h, the transfected cells were subjected to lysis using an immunoprecipitation assay buffer containing 10 mM Tris–HCl (7.5), 150 mM NaCl, 0.5 mM EDTA, 1 % Deoxycholate, and 1 % Triton X-100. Protein extracts were immunoprecipitated using anti-GFP beads (gtma-100, ChromoTek), and then the immunoprecipitates were extensively washed using an immunoprecipitation washing buffer containing 10 mM Tris–HCl (pH 7.5), 150 mM NaCl, 0.5 mM EDTA, and 0.05 % NP-40. Finally, the immunoprecipitated proteins were analyzed by Western blotting.

### 2.7 Proximity ligation assay (PLA)

At 24 h posttransfection, the transfected cells were fixed with 4% paraformaldehyde for 20 min at 37 °C, and then permeabilized with 0.1 % Triton X-100 in PBS for 8 min at room temperature. Following a PBS rinse, the cells were incubated with blocking solution (CAS-Block; Invitrogen) at room temperature for 30 min. Proximity ligation assay was performed using the Duolink® Proximity Ligation Assay kit (DUO92101, Sigma-Aldrich) according to manufacturer's protocols. Briefly, cell samples were incubated with anti-Hsp90 antibody (no. SC13119, Santa Cruz) and anti-FLAG antibody (no. F7425; Sigma-Aldrich) or anti-GFP antibody (no. G1544; Sigma-Aldrich) for 90 min. The probe incubation, ligation, and amplification were performed at 37 °C. The fluorescence signal of protein interactions was detected at an excitation of 594 nm and emission collected at 624 nm using the Leica SP5II confocal microscope (Leica) equipped with 63× oil immersion objective.

### 2.8 Statistical analysis

All data were expressed as means ± standard deviation (SD). For Western blot analysis, the Mann-Whitney *U* test was used to evaluate differences between samples. For analysis of confocal microscopic images, a two-sample *t*-test was used to evaluate differences between samples. All statistical analyses were conducted using SPSS software (version 18.0; IBM Corporation Armonk, NY), and the *p*-values less than 0.05 were considered statistically significant.

## 3 Results

### 3.1 Subcellular localization of VZV-encoded replication proteins

**Fig. 1:**
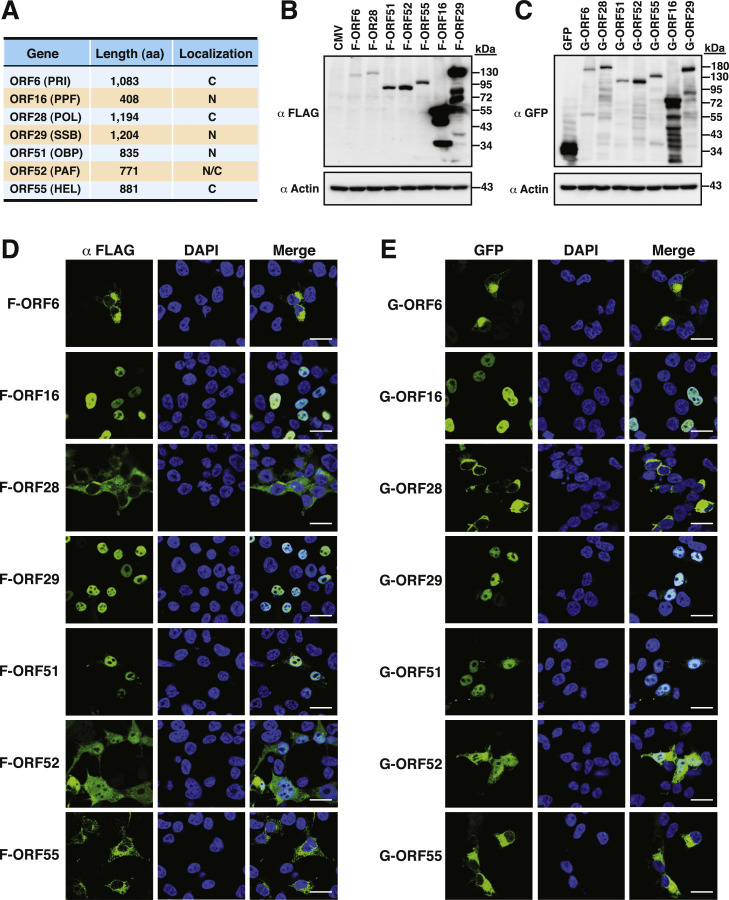
Expression and subcellular localization of VZV-encoded replication proteins in 293T cells. (A) Summary of the subcellular localization of seven VZV-encoded replication proteins. (B and C) Western blot analysis of the expression of FLAG-tagged and GFP-tagged replication proteins expressed in 293T cells. The experiments were performed twice independently with similar results. (D and E) Confocal microscopic analysis of the subcellular localization of FLAG-tagged and GFP-tagged replication proteins in 293T cells. Scale bars, 20 μm. The experiments were repeated at least three times with similar results.

Due to the lack of commercial antibodies against VZV replication proteins, we here constructed fusion proteins with a FLAG tag or a green fluorescent protein (GFP) tag to determine the subcellular localization of individual VZV replication proteins. To generate these expression plasmids, the viral DNA fragments corresponding to the coding regions of ORF6 (PRI), ORF16 (PPF), ORF28 (POL), ORF29 (SSB), ORF51 (OBP), ORF52 (PAF) and ORF55 (HEL) (Fig. 1A) were separately cloned into the expression vector pFLAG-CMV-2 (containing a coding sequence of a FLAG tag at the N-terminal end) or pEGFP-C2 (containing a coding sequence of a GFP tag at the N-terminal end). When these expression plasmids were individually transfected into 293T cells, Western blot analysis revealed that the molecular masses of F-ORF6, F-ORF16, F-ORF28, F-ORF29, F-ORF51, F-ORF52 and F-ORF55 were 125, 50, 135, 140, 86, 82 and 95 kDa, respectively (Fig. 1B), whereas the molecular masses of GFP-ORF6, GFP-ORF16, GFP-ORF28, GFP-ORF29, GFP-ORF51, GFP-ORF52 and GFP-ORF55 were 155, 80, 165, 170, 116, 112 and 125 kDa, respectively (Fig. 1C). All the constructed viral replication proteins migrated to their predicted positions in gels (Fig. 1B and 1C). In the confocal fluorescence microscopic experiments, we found that F-ORF16, F-ORF29 and F-ORF51 were localized in the nucleus of 293T cells (Fig. 1D). The ratios of nuclear to cytoplasmic fluorescence (Fn/c) of F-ORF16, F-ORF29 and F-ORF51 were up to 100 (Supplementary Fig. S1A). However, three replication proteins with enzymatic activities, including ORF6 (primase), ORF28 (DNA polymerase) and ORF55 (helicase), were restricted to the cytoplasm (Fig. 1D and Supplementary Fig. S1A, Fn/*c* < 0.2). Only F-ORF52 was distributed in both the nucleus and cytoplasm (Fig. 1D and Supplementary Fig. S1A, Fn/c of 1.5 ± 0.3). Consistently, the viral replication proteins carrying a GFP tag also exhibited the same subcellular localizations to their respective counterparts carrying a FLAG tag in 293T cells (Fig. 1E and Supplementary Fig. S1B).

### 3.2 ORF16 regulates the nuclear transport and the protein abundance of ORF28

**Fig. 2:**
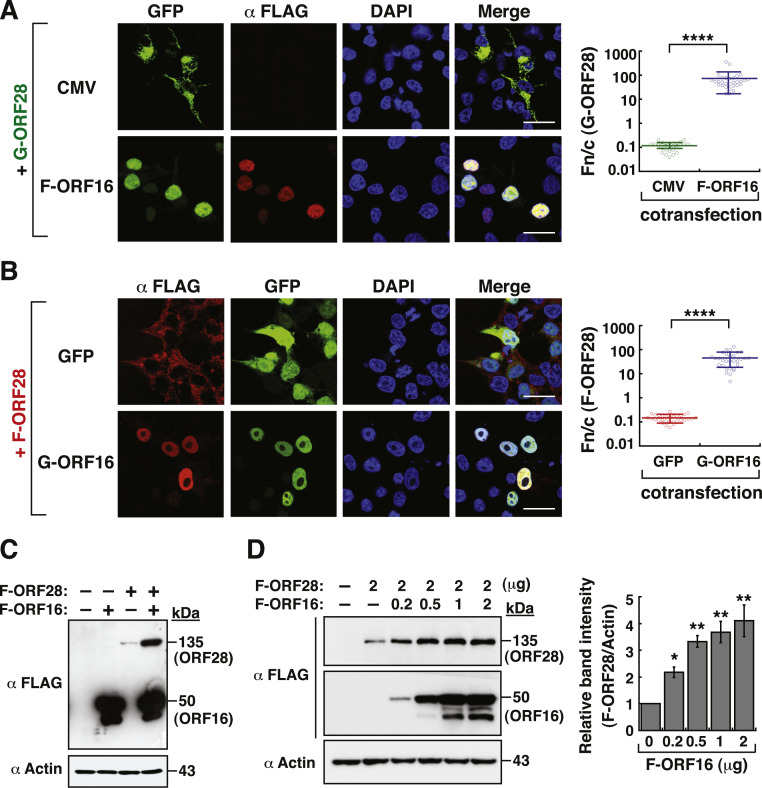
Effect of ORF16 on the nuclear transport and the protein abundance of ORF28. (A) Confocal microscopic images showing the subcellular localization of GFP-ORF28 in the presence or absence of F-ORF16 in 293T cells. Scale bars, 20 μm. The ratios of nuclear to cytoplasmic fluorescence (Fn/c) of GFP-ORF28 in individual cells for each cotransfection condition are shown in the right panel. Data are presented as means ± SD (*n* = 30 cells, three independent experiments). ****p < 0.0001 (two-sample *t*-test). (B) Confocal microscopic images showing the subcellular localization of F-ORF28 in the presence of GFP or GFP-ORF16 in 293T cells. Scale bars, 20 μm. The Fn/c values of F-ORF28 in individual cells for each cotransfection condition are shown in the right panel. Data are presented as means ± SD (*n* = 30 cells, three independent experiments). ****p < 0.0001 (two-sample *t*-test). (C) Western blot analysis of the protein lysates of 293T cells expressing F-ORF28, F-ORF16, or the combination of F-ORF28 and F-ORF16. (D) Effect of increasing amounts of F-ORF16 on the expression levels of F-ORF28 in 293T cells. Densitometry analysis of Western blots of F-ORF28 is shown in the right panel. Data are expressed as means ± SD from three independent experiments. * *p* < 0.05, ** *p* < 0.01 versus the group lacking F-ORF16 cotransfection (Mann-Whitney *U* test).

According to previous studies, POL enzymes of several herpesviruses need its interaction with their cognate PPFs for nuclear transport (Chen et al., 2023, 2005; Kawashima et al., 2013). Herein, we initially tested whether GFP-ORF28 could be conveyed into the nucleus by F-ORF16. Coexpression of GFP-ORF28 with F-ORF16 in 293T cells could lead to the nuclear transport of GFP-ORF28 (Fig. 2A, and Supplementary Fig. S2). Since several viral replication proteins including F-ORF29, F-ORF51, or the viral helicase-primase heterotrimeric subcomplex (containing F-ORF6, F-ORF52 and F-ORF55) could enter the nucleus (Fig. 1 and Supplementary Fig. S2), we also examined whether these viral proteins or subcomplex could mediate the nuclear entry of GFP-ORF28 in 293T cells. Confocal microscopic analysis showed that these viral replication proteins or subcomplex could not mediate the nuclear transport of GFP-ORF28 (Supplementary Fig. S2). To further support our findings, the subcellular localization of F-ORF28 was also analyzed in the presence of GFP or GFP-ORF16 (Fig. 2B). Consistently, the nuclear transport of F-ORF28 was mediated only by GFP-ORF16, but not GFP (Fig. 2B). Especially, when the protein levels of F-ORF16 and F-ORF28 were examined in co-transfected cells by Western blotting, we unexpectedly found that the protein levels of F-ORF28 were markedly increased by F-ORF16 in a dose-dependent manner (Fig. 2C and 2D). Besides coexpression with F-ORF16, coexpression with GFP-ORF16 also significantly increased the protein abundance of F-ORF28 as compared to coexpression with the GFP control (Supplementary Fig. S3A and S3C). These results suggest that ORF16 not only promotes the nuclear entry of ORF28 but also increases the protein abundance of ORF28.

To further determine whether the increased level of ORF28 by ORF16 was regulated at the transcriptional level, quantitative RT-PCR analysis was performed to determine ORF28 mRNA transcripts in cotransfected cells (Supplementary Fig. S3B and S3D). As compared to their respective control group, we found that neither F-ORF16 nor GFP-ORF16 enhanced the levels of ORF28 mRNA transcripts (Supplementary Fig. S3B and S3D), suggesting that the regulation of ORF28 abundance by ORF16 could be mediated through a post-transcriptional mechanism. Furthermore, when ORF16 was coexpressed with other replication proteins such as ORF6, ORF52 or ORF55 in 293T cells, we found that ORF16 specifically enhanced the protein abundance of F-ORF28, but not F-ORF6, F-ORF52 and F-ORF55 (Supplementary Fig. S3E).

### 3.3 Functional analysis of ORF16 deletion mutants

**Fig. 3:**
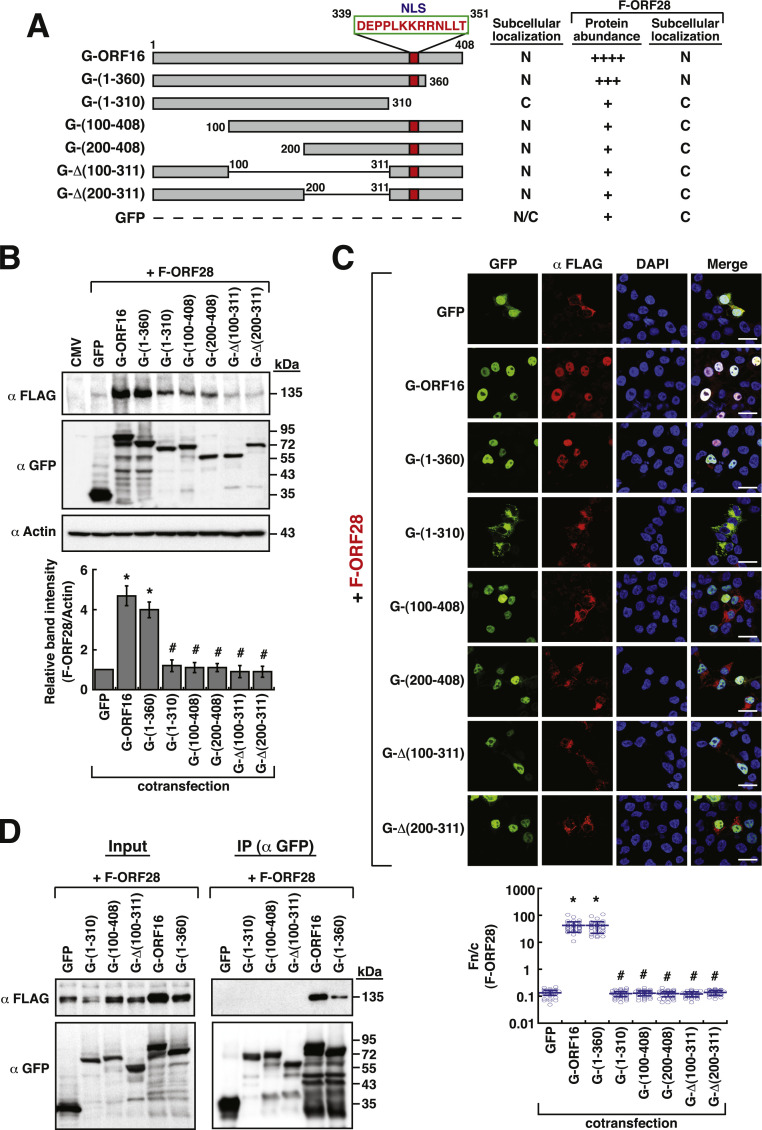
Effect of various GFP-ORF16 deletion mutants on the protein abundance and nuclear transport of F-ORF28. (A) Schematic diagram of GFP-ORF16 deletion mutants. An NLS is predicted in the region between aa 339 and 351 of ORF16. The subcellular localization of GFP-ORF16 deletion mutants and their ability to affect the protein abundance and nuclear transport of F-ORF28 are summarized in the diagram. (B) Western blot analysis of the expression levels of F-ORF28 in the presence of different GFP-ORF16 deletion mutants in 293T cells. Quantitative data of Western blots for F-ORF28 are expressed as means ± SD (*n* = 3) in the bottom panel. * *p* < 0.05 versus the GFP control group, # *p* < 0.05 versus the wild-type GFP-ORF16 group (Mann-Whitney *U* test). (C) Confocal microscopic analysis of the subcellular localization of F-ORF28 in the presence of different GFP-ORF16 deletion mutants in 293T cells. Scale bars, 20 μm. The Fn/c values of F-ORF28 in individual cells for each cotransfection condition were calculated and shown in the bottom panel. Data are presented as means ± SD (*n* = 30 cells, three independent experiments). * *p* < 0.0001 versus the GFP control group, # *p* < 0.0001 versus the wild-type GFP-ORF16 group (two-sample *t*-test). (D) Coimmunoprecipitation analysis of the interaction between F-ORF28 and different GFP-ORF16 deletion mutants in 293T cells. The F-ORF28 expression plasmid was cotransfected with the indicated GFP-ORF16 deletion mutants into 293T cells for 24 h. The protein lysates from these transfected cells were immunoprecipitated using anti-GFP antibody. The resultant immunoprecipitates were subjected to Western blot analysis using anti-FLAG or anti-GFP antibody. The experiments were repeated twice independently with similar results.

ORF16 is a 408-amino acid (aa) protein that contains a predicted nuclear localization signal (NLS) located in the region from aa 339 to 351 (Fig. 3 and Supplementary Fig. S4A). To determine the region of ORF16 required for the nuclear transport of ORF28 and for the enhancement of ORF28 abundance, different deletion constructs were generated in GFP-ORF16 (Fig. 3 and Supplementary Fig. S4A). These deletion mutants include two C-terminal deletion constructs such as GFP-ORF16(1–360) and GFP-ORF16(1–310), two N-terminal deletion constructs such as GFP-ORF16(100–408) and GFP-ORF16(200–408), and two internal deletion constructs such as GFP-Δ(100–311) and GFP-Δ(200–311) (Supplementary Fig. S4A and S4B). Confocal microscopic analysis revealed that only GFP-ORF16(1–310) was localized in the cytoplasm (Supplementary Fig. S4C, Fn/*c* < 0.2), whereas the other deletion constructs were localized in the nucleus (Supplementary Fig. S4C, Fn/*c* ≥ 100). When these deletion constructs were coexpressed with F-ORF28 in 293T cells, only GFP-ORF16(1–360) enhanced the protein abundance of F-ORF28 (Fig. 3A and 3B) and mediated the nuclear transport of F-ORF28 (Fig. 3A and 3C). However, the other GFP-ORF16 deletion mutants failed to enhance the protein level of F-ORF28 and to promote the nuclear transport of F-ORF28 (Fig. 3B and 3C). Coimmunoprecipitation analysis using anti-GFP antibody showed that F-ORF28 could be coimmunoprecipiated only by wild-type GFP-ORF16 and GFP-ORF16(1–360), but not other deletion mutants including GFP-ORF16(1–310), GFP-ORF16(100–408) and GFP-ORF16(100–311) (Fig. 3D). When a reciprocal coimmunoprecipitation was performed using anti-FLAG antibody, we also found that only wild-type GFP-ORF16 and GFP-ORF16(1–360), but not GFP-ORF16(1–310) or GFP, could be coimmunoprecipitated with F-ORF28 (Supplementary Fig. S5). However, due to the reasons that the amount of GFP-ORF16(1–310) was much lower than that of wild-type GFP-ORF16 and GFP-ORF16(1–360) in cell lysates used in coimmunoprecipitation assays (Fig. 3D and Supplementary Fig. S5), and both GFP-ORF16(1–310) and F-ORF28 were confined to the cytoplasm when coexpressed in cells (Fig. 3C), the results of the interaction between GFP-ORF16(1–310) and F-ORF28 still need further verification (see Discussion). Overall, our results suggested that the N-terminal 360-aa region of ORF16 is sufficient to interact with ORF28. The interaction between ORF16 and ORF28 could be critical for the nuclear transport of ORF28 and for the enhancement of ORF28 abundance.

### 3.4 The NLS-mutated ORF16 mutant retains the ability to increase the abundance of ORF28

**Fig. 4:**
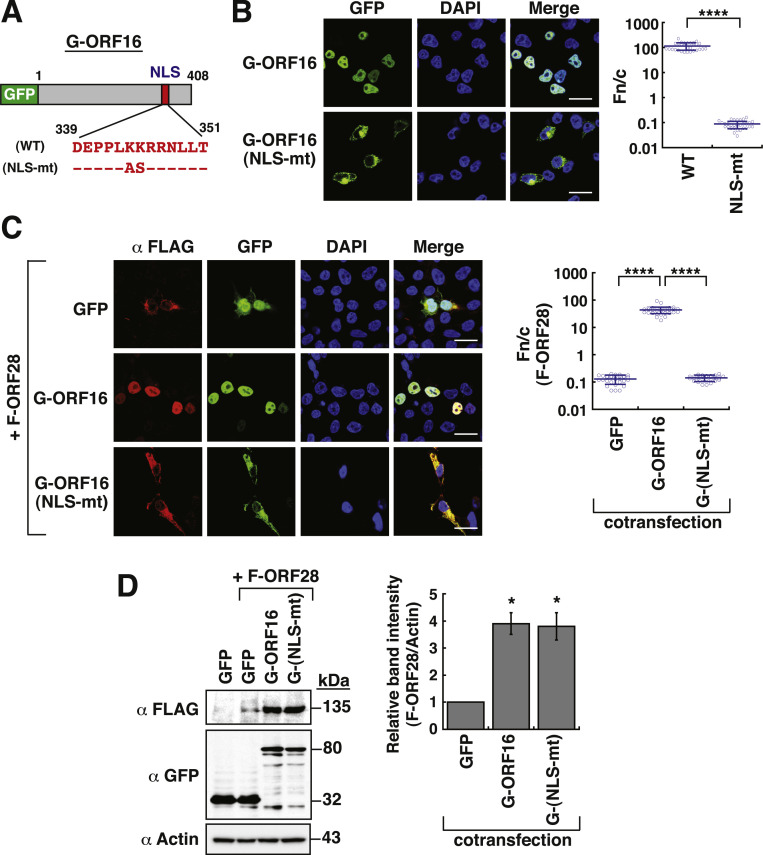
The NLS-defective GFP-ORF16 mutant retains a normal ability to enhance F-ORF28 abundance. (A) Schematic diagram of wild-type and NLS-defective GFP-ORF16. Specific mutations at the predicted NLS of GFP-ORF16 are shown in the diagram. (B) Confocal microscopic analysis of the subcellular localization of wild-type and NLS-defective GFP-ORF16 in 293T cells. Scale bars, 20 μm. The Fn/c values of GFP-ORF16 and its NLS-defective mutant in individual cells were calculated and shown in the right panel. Data are presented as means ± SD (*n* = 30 cells, three independent experiments). **** *p* < 0.0001 (two-sample *t*-test). (C) Confocal microscopic analysis of the subcellular localization of F-ORF28 and GFP-ORF16(NLS-mt) in 293T cells. Scale bars, 20 μm. The Fn/c values of F-ORF28 in individual cells in the indicated cotransfections were calculated and shown in the right panel. Data are presented as means ± SD (*n* = 30 cells, three independent experiments). **** *p* < 0.0001 (two-sample *t*-test). (D) Representative Western blot images and densitometry analysis of F-ORF28 in the presence of GFP, GFP-ORF16 or GFP-ORF16(NLS-mt). Quantitative data of Western blots for F-ORF28 are presented as means ± SD (*n* = 3). * *p* < 0.05 versus the GFP control group (Mann-Whitney *U* test).

Next, we investigated whether an ORF16 mutant that is defective in nuclear transport could enhance the protein abundance of ORF28 in the cytoplasm. In this regard, we mutated basic residues in the predicted NLS motif of ORF16 (Fig. 4A, NLS-mt). Confocal microscopic analysis displayed that the resultant GFP-ORF16(NLS-mt) mutant could not enter the nucleus (Fig. 4B, Fn/*c* < 0.2). To further determine whether the predicted NLS-like motif from aa 339 to 351 (amino acid residues: DEPPLKKRRNLLT) was a functional NLS in ORF16, the 13-aa motif was fused to GFP at the C-terminal end or fused to 3 copies of GFP (3×GFP) at the N-terminal end (Supplementary Fig. S6A and S6B). Additionally, a mutated NLS-like motif (NLSmt) that contains amino acid substitutions K444A and K445S was also fused to GFP or 3×GFP. The resulting fusion constructs were designated as GFP-NLS and GFP-NLSmt (Supplementary Fig. S6A), as well as NLS-3×GFP and NLSmt-3×GFP (Supplementary Fig. S6B). Confocal microscopic analysis revealed that GFP-NLS, but not GFP-NLSmt, strongly accumulated in the nucleus (Supplementary Fig. S6A, Fn/c of 10.2 versus Fn/c of 1.6). Similarly, NLS-3×GFP, but not NLSmt-3×GFP, also showed dominant nuclear accumulation in transfected cells (Supplementary Fig. S6B, Fn/c of 74.5 versus Fn/c of 0.09). These results demonstrated that the predicted NLS-like motif from aa 339 to 351 of ORF16 is a functional NLS.

Next, we examined the effect of the NLS-deficient GFP-ORF16 mutant on the subcellular localization and protein abundance of F-ORF28 in 293T cells. When GFP-ORF16(NLS-mt) was coexpressed with F-ORF28 in 293T cells, we found that both proteins appeared to be colocalized in the cytoplasm (Fig. 4C). Importantly, Western blot analysis showed that GFP-ORF16(NLS-mt), like wild-type GFP-ORF16, still retained a normal ability to enhance the protein abundance of F-ORF28 (Fig. 4D).

### 3.5 The nuclear localization of ORF28 may not be the key determinant in changing its protein abundance

**Fig. 5:**
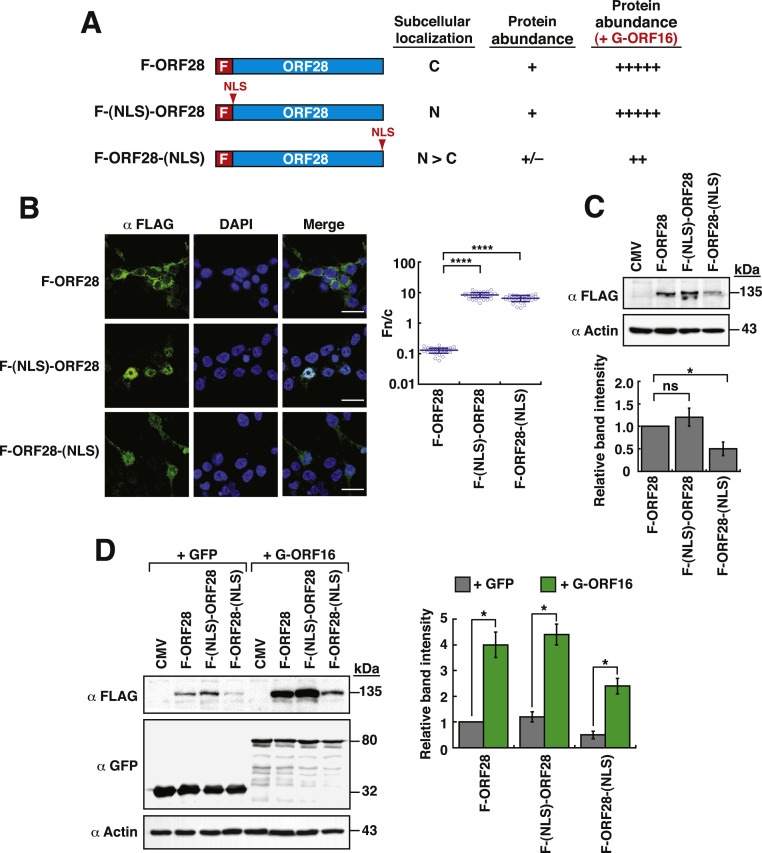
Addition of an NLS to ORF28 increases nuclear accumulation, but not protein abundance. (A) Schematic diagram of F-ORF28 constructs with an NLS motif at the N-terminal or C-terminal end. The subcellular localization and protein abundance of the indicated constructs are summarized. (B) Confocal microscopic analysis of the subcellular localization of F-ORF28, F-(NLS)-ORF28 and F-ORF28-(NLS) in 293T cells. Scale bars, 20 μm. The Fn/c values of F-ORF28, F-(NLS)-ORF28 and F-ORF28-(NLS) in individual cells were calculated and shown in the right panel. Data are presented as means ± SD (*n* = 30 cells, three independent experiments). **** *p* < 0.0001 (two-sample *t*-test). (C) Western blot analysis of the expression of F-ORF28, F-(NLS)-ORF28 or F-ORF28-(NLS) alone in 293T cells. Densitometry analysis of Western blots of F-ORF28, F-(NLS)-ORF28 and F-ORF28-(NLS) is shown in the bottom panel. Data are expressed as means ± SD (*n* = 3). * *p* < 0.05, ns = not significant (Mann-Whitney *U* test). (D) Western blot analysis of the expression levels of F-ORF28, F-(NLS)-ORF28 and F-ORF28-(NLS) in the presence of GFP or GFP-ORF16. Densitometry analysis of Western blots of F-ORF28, F-(NLS)-ORF28 and F-ORF28-(NLS) in each condition is shown in the right panel. Data are expressed as means ± SD from three independent experiments. * *p* < 0.05 (Mann-Whitney *U* test).

As described above, the interaction between GFP-ORF16 and F-ORF28 in either the cytoplasm or the nucleus could enhance the protein abundance of F-ORF28. To further investigate whether the nuclear localization of ORF28 itself could increase its protein abundance, a functional NLS derived from the SV40 T antigen was inserted into the N-terminal or C-terminal end of F-ORF28 (Fig. 5A). As expected, the resultant constructs, F-(NLS)-ORF28 and F-ORF28-(NLS), were mainly localized in the nucleus (Fig. 5B). The Fn/c values of F-ORF28, F-(NLS)-ORF28 and F-ORF28-(NLS), were 0.1, 8.4 and 6.5, respectively. However, the protein levels of these two NLS-containing constructs expressed in 293T cells were not increased significantly as compared to that of the parental F-ORF28 (Fig. 5C). When F-(NLS)-ORF28 or F-ORF28-(NLS) was coexpressed with GFP-ORF16, we found that the protein levels of both NLS-containing constructs could be substantially enhanced by GFP-ORF16 (Fig. 5D). These results suggested that the functional interaction between ORF28 and ORF16 could be the key determinant in contributing to the enhancement of ORF28 abundance.

### 3.6 The low abundance of ORF28 itself in cells is due to rapid protein degradation via the ubiquitin-proteasome system, but not via the autophagic-lysosomal pathway

**Fig. 6:**
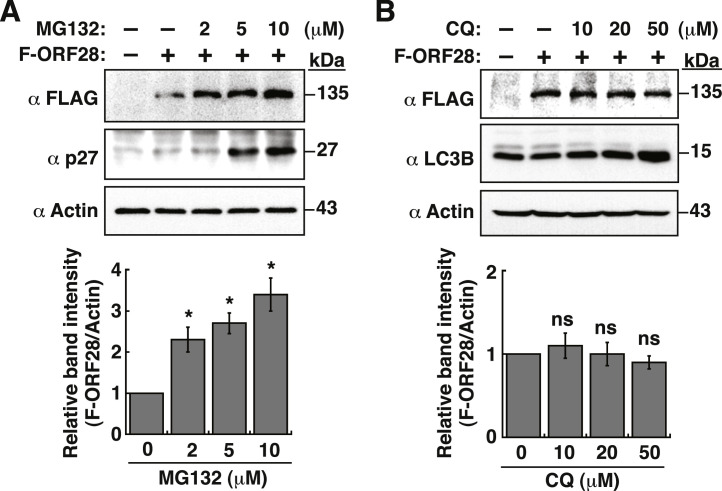
The low-level expression of ORF28 in cells is due to its rapid protein degradation mediated by the ubiquitn-proteaosmal system, but not by the autophagic pathway. (A) Effect of increasing amounts of MG132 on the protein abundance of F-ORF28. 293T cells were transfected with pCMV-F-ORF28 for 24 h, and then treated with different concentrations of a proteasome inhibitor MG132. The protein levels of F-ORF28 and p27 in transfected cells were analyzed by Western blotting. Quantitative analysis of Western blots for F-ORF28 in each condition is shown in the bottom panel. Data are presented as means ± SD from three independent experiments. * *p* < 0.05 versus the untreated group (Mann-Whitney *U* test). (B) Effect of increasing amounts of chloroquine on the protein abundance of F-ORF28. 293T cells were transfected with pCMV-F-ORF28 for 24 h, and then treated with different concentrations of an autophagic inhibitor chloroquine (CQ) for another 4 h. The protein levels of F-ORF28 and LC3B in transfected cells were analyzed by Western blotting. Quantitative analysis of Western blots for F-ORF28 in each condition is shown in the bottom panel. Data are presented as means ± SD from three independent experiments. Statistical analysis was performed between CQ-treated groups and the untreated group (ns, not significant; Mann-Whitney *U* test).

Since both the ubiquitin-proteasome system and autophagy are two major pathways for protein degradation (Li et al., 2022), we investigated whether these two pathways were involved in the regulation of ORF28 abundance. To test these possibilities, 293T cells were transfected with pCMV-F-ORF28 for 24 h, and then treated with different concentrations of a proteasome inhibitor MG132 (Lee et al., 2021; Zhu et al., 2016) or an autophagic inhibitor chloroquine (Redmann et al., 2017) for another 4 h. Western blot analysis showed that the protein level of F-ORF28 could be significantly enhanced by MG132, but not by chloroquine (Fig. 6A and 6B). In these experiments, the cyclin-dependent kinase inhibitor p27 was used as a positive control of MG132 treatment (Tsvetkov et al., 1999), whereas LC3B served as a positive control of chloroquine treatment (Sharifi et al., 2015) (Fig. 6A and 6B). These results suggested that the low level of F-ORF28 maintained in cells is mainly due to rapid protein degradation mediated by the ubiquitin-proteasome system.

### 3.7 A Hsp90 inhibitor radicicol can disrupt the interaction between ORF28 and ORF16

**Fig. 7:**
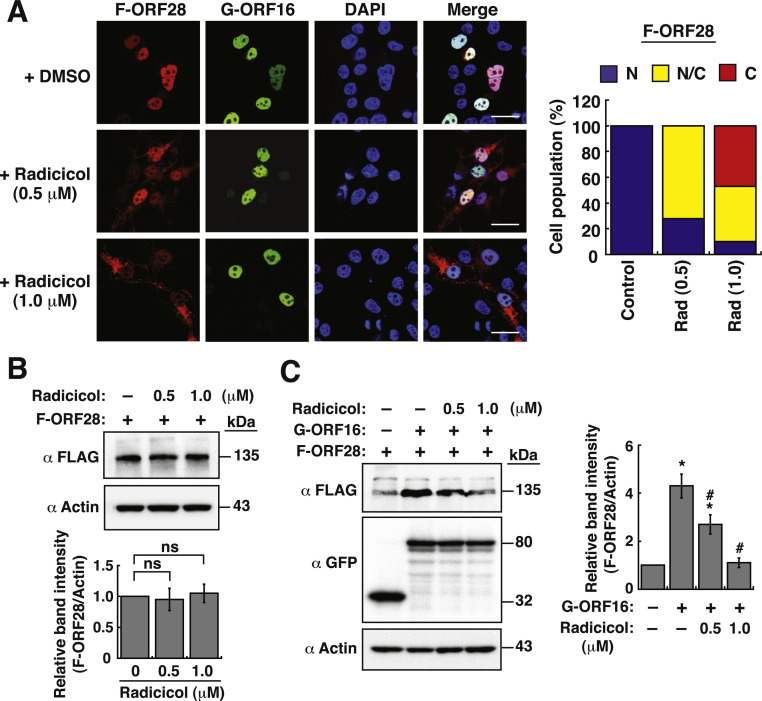
Radicicol can disrupt the interaction between ORF28 and ORF16. (A) Effect of increasing amounts of radicicol on the subcellular localization of F-ORF28 and GFP-ORF16 in 293T cells. In the experiments, 293T cells were cotransfected with pCMV-F-ORF28 and pCMV-GFP-ORF16, and then treated with DMSO (as a solvent control) or radicicol (0.5 and 1.0 μM) for 24 h. The subcellular localization of F-ORF28 and GFP-ORF16 in transfected cells was analyzed by confocal microscopy. Scale bars, 20 μm. As noted, radicicol did not affect the nuclear import of GFP-ORF16. The subcellular localization patterns of F-ORF28 in different treatment groups were calculated from three independent experiments (*n* = 85 cells) and plotted in the right panel. N, nucleus (Fn/*c* > 5); N/C, both nucleus and cytoplasm (0.2 < Fn/*c* < 5); C, cytoplasm (Fn/*c* < 0.2). (B) Effect of radicicol on the protein level of F-ORF28 in 293T cells. Quantitative analysis of Western blots for F-ORF28 in each condition is shown in the bottom panel. Data are expressed as means ± SD from three independent experiments (ns, not significant; Mann-Whitney *U* test). (C) Effect of radicicol on the protein level of F-ORF28 in 293T cells cotransfected with GFP-ORF16. Quantitative analysis of Western blots for F-ORF28 expressed in the indicated conditions is shown in the right panel. Data are presented as means ± SD from three independent experiments. * *p* < 0.05 versus the group lacking GFP-ORF16 cotransfection, # *p* < 0.05 versus the untreated GFP-ORF16-cotransfected group (Mann-Whitney *U* test).

Hsp90 is an important chaperone in cells (Biebl and Buchner, 2019; Schopf et al., 2017; Theodoraki and Caplan, 2012). Previous studies have shown that Hsp90 could be involved in the interaction between POLs and their cognate PPFs of several herpesviruses (Chen et al., 2023; Kawashima et al., 2013). To determine whether Hsp90 was required for the interaction between ORF28 and ORF16, a Hsp90 inhibitor radicicol at 0.5 or 1.0 μM (Chen et al., 2023; Kawashima et al., 2013) was used to treat 293T cells co-transfected with pCMV-F-ORF28 and pCMV-GFP-ORF16. Confocal microscopic analysis found that treatment with radicicol significantly prevented the nuclear transport of F-ORF28 in a dose-dependent manner (Fig. 7A). However, radicicol treatment did not affect the nuclear import of GFP-ORF16 (Fig. 7A). When the protein abundance of F-ORF28 was examined by Western blotting, we found that radicical could not affect expression level of F-ORF28 alone (Fig. 7B). However, the increased levels of F-ORF28 by GFP-ORF16 could be significantly reduced by radicicol (Fig. 7C). These results suggested that radicicol could block the interaction between F-ORF28 and GFP-ORF16, thereby resulting in the cytoplasmic retention and low abundance of F-ORF28.

**Fig. 8:**
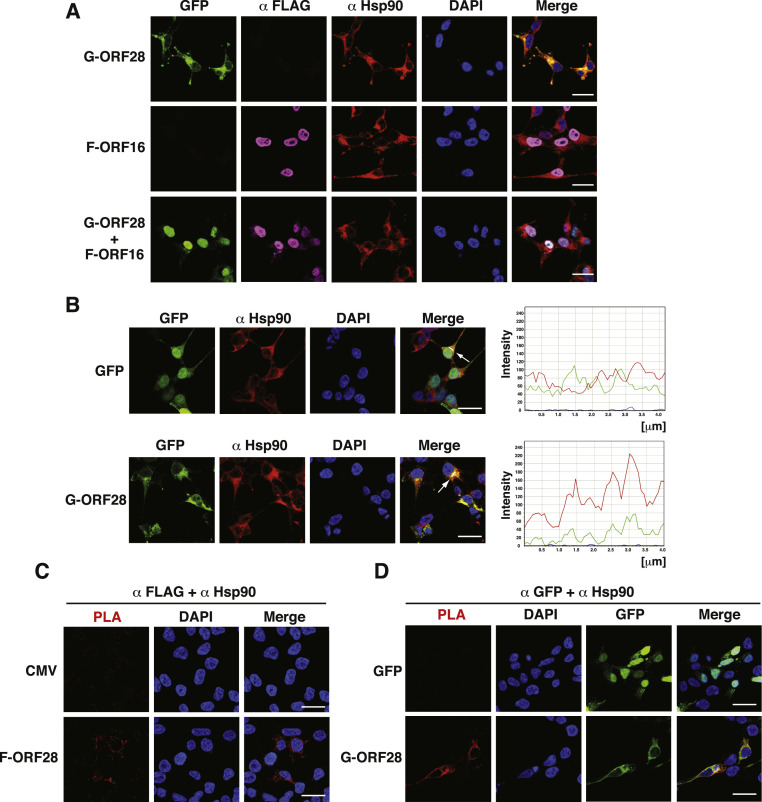
ORF28 colocalizes with Hsp90 in the cytoplasm of 293T cells. (A) Confocal microscopic analysis of the subcellular localization of GFP-ORF28, F-ORF16 and Hsp90 in the indicated transfected cells. Scale bars, 20 μm. (B) Evaluation of the interaction between ORF28 and Hsp90 in 293T cells by the intensity-based correlation analysis. Arrows in merged images indicate cells selected for the intensity correlation analysis. Scale bars, 20 μm. (C) Evaluation of the interaction between F-ORF28 and Hsp90 in 293T cells by PLA using both anti-FLAG and anti-Hsp90 antibodies. Red puncta represent PLA positive signals. Scale bars, 20 μm. (D) Evaluation of the interaction between GFP-ORF28 and Hsp90 in 293T cells by PLA using both anti-GFP and anti-Hsp90 antibodies. Red puncta represent PLA positive signals. Scale bars, 20 μm.

To examine the potential role of Hsp90 in the interaction between ORF28 and ORF16, the subcellular localization of GFP-ORF28, F-ORF16 and Hsp90 was analyzed by confocal fluorescence microscopy. We found that Hsp90 was mainly localized in the cytoplasm and the subcellular localization of Hsp90 was not affected by the expression of GFP-ORF28, F-ORF16 or both GFP-ORF28 and F-ORF16 (Fig. 8A). However, we noticed that in GFP-ORF28-transfected cells, GFP-ORF28 appeared to be colocalized with Hsp90 in the cytoplasm (Fig. 8A). To further support our observation, we performed the intensity-based correlation analysis and proximity ligation assay (PLA) to evaluate the potential interaction between F-ORF28 and Hsp90. PLA is a sensitive tool to detect the protein-protein interaction in situ when target proteins are present within the required proximity (distance < 40 nm) (Alam, 2018). This assay principle is that, if two target proteins interact, the interaction between the proteins can be recognized by specific primary antibodies and by corresponding secondary antibodies conjugated with a single-stranded oligonucleotide. In the presence of connector oligonucleotides and ligase, the DNA oligonucleotide forms a circle. The circular DNA can be amplified by DNA polymerase, and the amplified DNA is detected with a fluorescent probe. PLA signals are visualized by fluorescence microscopy as punctate spots.

Based on the intensity correlation analysis, there was a linear relationship between the fluorescence intensities of F-ORF28 and Hsp90 (Fig. 8B). When PLA experiments were performed using anti-FLAG and anti-Hsp90 antibodies, we also found that punctate PLA signals were observed in F-ORF28-transfected cells, but not in the empty vector-transfected cells (Fig. 8C). Similarly, when PLA experiments were carried out using anti-GFP and anti-Hsp90 antibodies in GFP-transfected cells and in GFP-ORF28-transfected cells, only GFP-ORF28-transfected cells showed PLA-positive signals (Fig. 8D). All these results suggested the interaction between F-ORF28 and Hsp90 in the cytoplasm.

## 4 Discussion

Compared to other human herpesviruses, the interaction and assembly of the essential DNA replication proteins of VZV remains largely unknown. In this study, we initially examined the subcellular localization of seven VZV-encoded replication proteins individually expressed in cells. According to the prediction of NLSs in these viral proteins using cNLS Mapper program (https://nls-mapper.iab.keio.ac.jp/cgi-bin/NLS_Mapper_form.cgi) (Kosugi et al., 2009), only three VZV replication proteins including ORF16 (PPF), ORF29 (SSB) and ORF51 (OBP) possess NLS motifs (Supplementary Fig. S7). In the prediction, a monopartite NLS was found between aa 339 and 351 of ORF16, whereas a bipartite NLS was found in the region from aa 1175 to 1200 of ORF29 and in the region from aa 31 to 54 of ORF51 (Supplementary Fig. S7). Despite the fact that the NLS prediction of ORF29 was inconsistent with previous research (Stallings and Silverstein, 2005), our results show that ORF16, ORF29 and ORF51 have strong nuclear accumulation independently of other viral proteins (Fig. 1). Although VZV replication occurs in the cell nucleus, we here observed that three viral proteins including ORF28 (POL), ORF6 (PRI) and ORF55 (HEL) were mainly localized in the cytoplasm (Fig. 1). Noteworthily, unlike POL homologs from HSV-1 and HCMV (UL30 and UL54, respectively), the VZV ORF28 lacks a functional NLS and cannot move into the nucleus by itself. The cytoplasmic restriction of specific viral replication components in cells may be an important regulatory mechanism to prevent untimely viral DNA replication in the nucleus when other essential replication components are not yet available or when leaky expression of these viral proteins is induced under certain stressful conditions. By using different combinations of viral replication proteins coexpressed in cells, we demonstrated that the nuclear transport of ORF28 can be mediated by ORF16 (forming a heterodimeric subcomplex), whereas the nuclear import of both ORF6 and ORF55 requires their association with ORF52 (forming a heterotrimeric subcomplex) (Fig. 1 and Supplementary Fig. S2). Currently, the detailed pathway of nuclear import of several VZV replication components still remains to be determined. According to the previous studies on the VZV ORF29, the nuclear transport of the protein is mediated through the classical importin α/β pathway (Stallings and Silverstein, 2005). Moreover, since ORF16 contains a monopartite NLS which matches the consensus for classical NLSs binding to importin α/β (Harreman et al., 2004), it is therefore possible that the nuclear import of ORF16 relies on the importin α/β-mediated pathway, similar to what reported from its homologues from HSV-1, HCMV and Pseudorabies virus (Alvisi et al., 2008, 2005; Wang et al., 2016). In future, specific inhibitors of the importin α/β-mediated nuclear import pathway, such as Ivermectin, Mifepristone and Bimax2 (Alvisi et al., 2023; Cross et al., 2024; Kosyna and Depping, 2018; Raza et al., 2020), will be first used in our assay system to delineate the transport process of specific VZV replication proteins or subcomplexes. Understanding the role of the importin α/β heterodimer or other cellular proteins (such as protein kinases for post-transcriptional modifications or chaperones for protein folding) in nuclear transport of VZV replication components may be helpful to develop new strategies against VZV infection.

Besides promoting the nuclear transport of ORF28, ORF16 could also increase the protein abundance of ORF28 (Fig. 2). Due to the limited information provided by other herpesviruses, we currently do not know whether this event regarding the enhancement of POL levels by PPF is conserved for all herpesviruses or is only unique to some herpesviruses such as VZV. It should be noted that although all herpesviral DNA polymerase processivity factors (such as HSV-1 UL42, HCMV UL44, EBV BMRF1 and KSHV ORF59) share structural homology and functional similarity, these proteins exhibit only low amino acid identity (Cohan and Frappier, 2021). For example, only 25 % amino acid identity could be detected between HSV-1 UL42 and VZV ORF16. Furthermore, several studies have shown that the interactions between POLs and their cognate PPFs of herpesviruses were specific, and they could not be functionally replaced by other POLs and PPFs of different herpesviruses (Bruce et al., 2009; Lin et al., 1998). Deletion analysis of ORF16 has revealed that the N-terminal 360-aa region, but not the N-terminal 310-aa region, is sufficient for the interaction with ORF28, for the nuclear import of ORF28, and for the enhancement of ORF28 abundance (Fig. 3). However, since the amount of GFP-ORF16(1–310) in cell samples used in coimmunoprecipitation assays was much lower than that of wild-type GFP-ORF16 and GFP-ORF16(1–360) in cell samples (Fig. 3D and Supplementary Fig. S5), we could not completely rule out that the failure to detect the interaction between GFP-ORF16(1–310) and F-ORF28 was due to the low amount of the protein. When the protein structure of VZV ORF16 was predicted and aligned with known template structures using Phyre2 system (http://www.sbg.bio.ic.ac.uk/phyre2/html/page.cgi) (Kelley et al., 2015), we found that the protein region from aa 22 to 315 of VZV ORF16 was structurally similar to the HSV-1 UL42 protein region from 28 to 317 (Supplementary Fig. S8). Noteworthily, the N-terminal 315-aa region of HSV-1 UL42 has previously reported as a minimal domain essential for the interaction with its DNA polymerase (Tenney et al., 1993). Due to the structural similarity between VZV ORF16 and HSV-1 UL42 (Supplementary Fig. S8), we speculate that a small motif (3∼5 amino acid residues) downstream of the N-terminal 310-aa region of VZV ORF16 may play a critical role in the interaction with ORF28, perhaps participating in the direct binding or helping shape the structure of the protein. In future, the minimal domain of ORF16 essential for the interaction with ORF28 still needs to be further explored. Additionally, although the NLS-defective ORF16 failed to convey ORF28 into the nucleus, the mutant could still increase ORF28 abundance (Fig. 4). Our findings therefore propose that the interaction between ORF16 and ORF28 is the key determinant for enhancing ORF28 abundance. Consistent with this notion, adding a functional NLS to the N-terminal or C-terminal end of ORF28, albeit promoting nuclear accumulation of proteins, did not significantly increase the levels of the constructed proteins (Fig. 5).

As compared to other VZV replication components, we noticed that ORF28 was expressed at a relatively low level in transfected cells (Fig. 1). The low level of ORF28 in transfected cells was mainly due to rapid protein degradation mediated by the ubiquitin-proteasome system, but not by the autophagy-lysosomal pathway (Fig. 6). At present, mapping the interaction domain of ORF28 with ORF16 is ongoing. To date, only two DNA polymerases of herpesviruses (including HSV-1 and HCMV) have been characterized for their PPF-interacting domain (Alvisi et al., 2006; Digard et al., 1993; Digard and Coen, 1990; Loregian et al., 2003). These studies demonstrated that only a small C-terminal region of herpesviral DNA polymerases is sufficient to interact with their PPFs. It will be interesting to see whether the C-terminal region of VZV ORF28 possesses the conserved function in interaction with ORF16.

Hsp90 is a key component of chaperone networks that assist protein folding and prevent protein aggregation (Biebl and Buchner, 2019; Schopf et al., 2017; Theodoraki and Caplan, 2012). We here showed that inhibition of Hsp90 activity by radicicol significantly blocks the nuclear transport of ORF28, but not ORF16 (Fig. 7), suggesting that Hsp90 is involved in the interaction between ORF16 and ORF28. Consistently, Hsp90 has previously been shown to play a critical role in the interaction of POL enzymes and PPF subunits of EBV and KSHV (Chen et al., 2023; Kawashima et al., 2013). Additionally, previous studies also demonstrated that Hsp90 participates in nuclear import of the HSV-1 POL enzyme (Burch and Weller, 2005). Since VZV ORF28 is a foreign protein containing over 1000 amino acids, a correct folding of this protein after synthesis may be important for its interaction with ORF16. In this case, Hsp90 may serve as a main chaperone to maintain the three-dimensional structure of ORF28 and to assist the subsequent interaction between ORF28 and ORF16. In fact, colocalization of ORF28 and Hsp90 in the cytoplasm could be observed in the confocal fluorescence microscopic experiments and PLA assays (Fig. 8). Although we observed colocalization of ORF28 and Hsp90 in the cytoplasm, we failed to demonstrate the interaction of ORF28 with endogenous or exogenous Hsp90 by coimmunoprecipitation assays (data not shown). It could be possible that the interaction between ORF28 and Hsp90 in cells is weak and transient. Due to the fact that Hsp90 inhibition is also known to inhibit nuclear location of other VZV replication protein ORF29 (Kyratsous and Silverstein, 2007) and to attenuate VZV replication in cell culture (Kyratsous and Silverstein, 2007), Hsp90 may be a promising therapeutic target against VZV infection.

In summary, this study demonstrates that several VZV-encoded replication enzymes *per se* are localized in the cytoplasm. Additionally, we show that the VZV PPF subunit (ORF16) can promote both the nuclear entry and the abundance of the POL enzyme (ORF28). Furthermore, we propose a critical role of Hsp90 in assisting the interaction between ORF28 and ORF16.

## Funding

This work was supported by medical research grants CMRPG6D0191, CMRPD6L0013 and BMRP921 from Chang Gung Memorial Hospital at Chiayi and by the grant 112–2320-B-182–037-MY3 from the National Science and Technology Council of Taiwan.

## CRediT authorship contribution statement

**Huang-Shen Lin:** Writing – original draft, Methodology, Investigation, Funding acquisition, Formal analysis, Conceptualization. **Cheng-Han Li:** Methodology, Investigation, Formal analysis. **Lee-Wen Chen:** Investigation, Data curation. **Shie-Shan Wang:** Methodology, Formal analysis. **Li-Yu Chen:** Validation, Investigation. **Chien-Hui Hung:** Validation, Resources, Methodology. **Chun-Liang Lin:** Resources, Formal analysis, Conceptualization. **Pey-Jium Chang:** Writing – review & editing, Writing – original draft, Supervision, Resources, Funding acquisition, Conceptualization.

## Declaration of competing interest

The authors declare that they have no known competing financial interests or personal relationships that could have appeared to influence the work reported in this paper.

## Data Availability

Data will be made available on request.

## Appendix Supplementary materials

Image, application 1
