# Supplementary material for: The varicella-zoster virus ORF16 protein promotes both the nuclear transport and the protein abundance of the viral DNA polymerase subunit ORF28
Source: Virus Res. 2024 Apr 26;345:199379. doi: 10.1016/j.virusres.2024.199379 (PMC11061344; doi:10.1016/j.virusres.2024.199379)
Supplement: Supplementary file 1 [file mmc1.pdf]

Supplementary Materials

Table of Contents

1. **Supplementary Figures**……………………………………………………………2

Fig. S1. Quantification of the nuclear to cytoplasmic fluorescence ratio (Fn/c) of FLAG-tagged and GFP-tagged VZV replication proteins in cells………………………………2

Fig. S2. Effects of coexpression of specific VZV replication proteins on subcellular localization of GFP-ORF28…………………………………………………………...3

Fig. S3. ORF16 increses ORF28 in a transcription-independent manner …………………4

Fig. S4. Expression and subcellular localization of wild-type and mutant GFP-ORF16……………………………………………………………………………………6

Fig. S5. Coimmunoprecipitation analysis of the interaction between F-ORF28 and GFP-ORF16 deletion mutants……………………………………………………………………7

Fig. S6. The basic amino acid motif ([339]DEPPLKKRRNLLT[351]) of ORF16 is a functional nuclear localization signal……………………………………………………………..8

Fig. S7. Schematic diagram of VZV replication proteins and the predicted NLSs………..9

Fig. S8. Structural predication of VZV ORF16 using Phyre2 system……………………10

2. **Supplementary Methods and Materials**……………………………………………11

2.1. Quantitative reverse transcription-PCR (RT-qPCR)……………………………...11

2.2. Coimmunoprecipitation with an anti-FLAG antibody……………………………12

2.3. NLS prediction using cNLS Mapper program……………………………………13

2.4. Protein stucture prediction using Phyre2 system…………………………………13

3. **Supplementary References** ……………………………………………………………13

1. Supplementary Figures

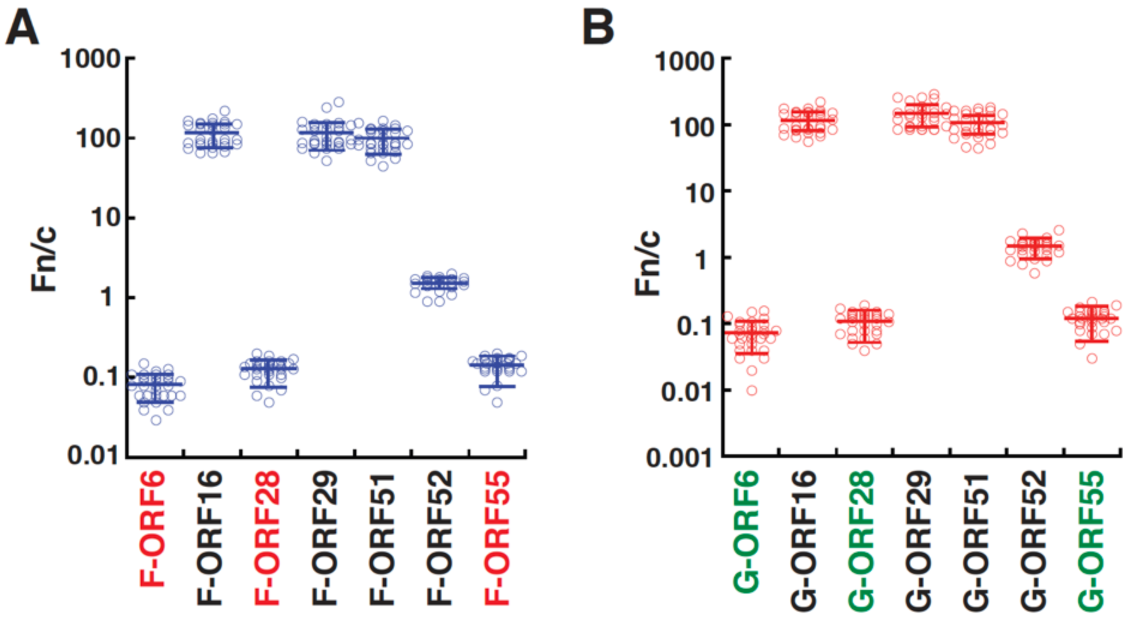

Supplementary Fig. S1. Quantification of the nuclear to cytoplasmic fluorescence ratio (Fn/c) of FLAG-tagged and GFP-tagged VZV replication proteins in cells. The Fn/c values of FLAG-tagged (A) and GFP-tagged VZV replication proteins (B) in 293T cells were determined from digitized confocal images. Data are presented as means ± SD scored from 30 cells from three independent experiments. As noted, three VZV replication proteins including ORF6, ORF28 and ORF55 are mainly localized in the cytoplasm (Fn/c < 0.2).

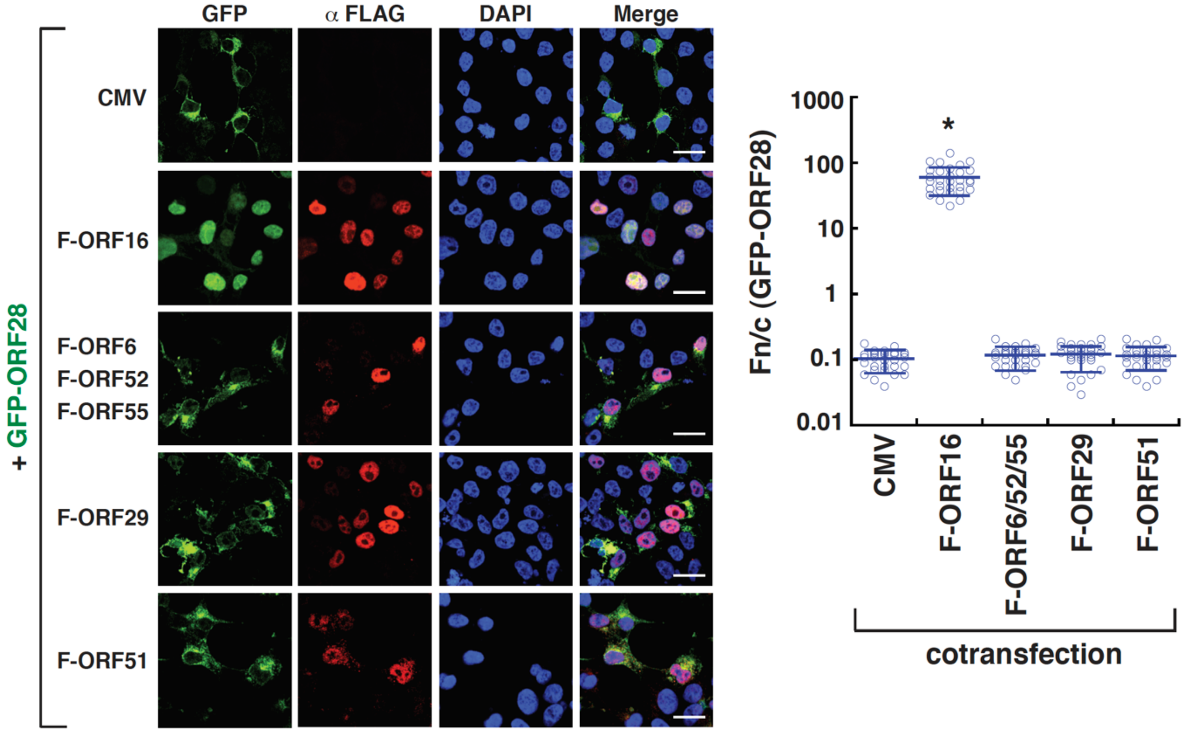

Supplementary Fig. S2. Effects of coexpression of specific VZV replication proteins on subcellular localization of GFP-ORF28. Confocal microscopic analysis was performed to determine the subcellular localization of GFP-ORF28 when coexpressed with F-ORF16, the helicase-primase heterotrimeric subcomplex (containing F-ORF6, F-ORF52 and F-ORF55), F-ORF29 or F-ORF51 in 293T cells (left panel). Scale bars, 20 μm. The Fn/c values of GFP-ORF28 in the indicated co-transfected cells were calculated and shown in the right panel. Data are presented as means ± SD ($n = 30$ cells, three independent experiments). * $p < 0.0001$ versus the empty vector (CMV) control (two-sample t-test).

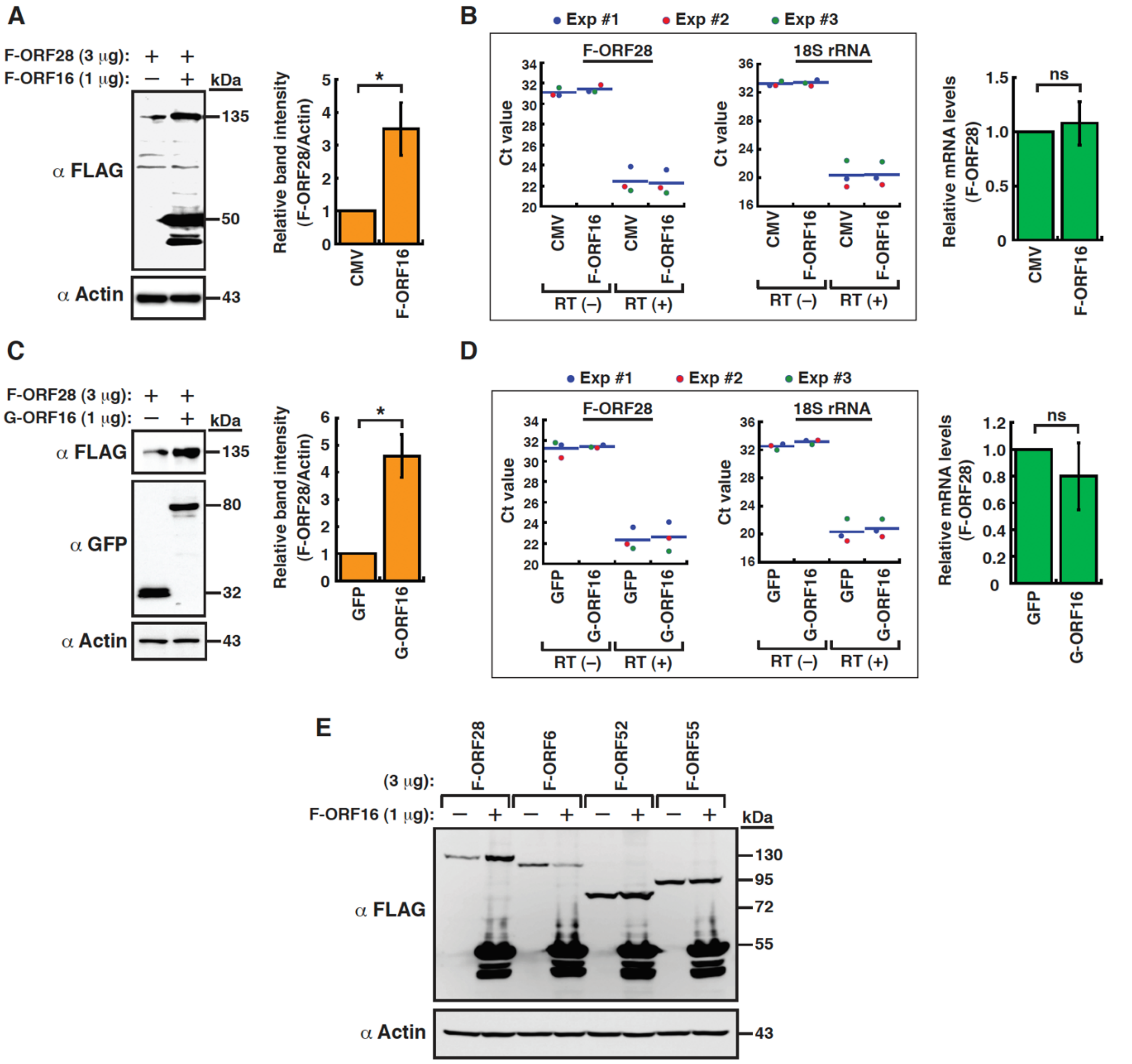

Supplementary Fig. S3. ORF16 increses ORF28 in a transcription-independent manner. (A) Representative Western blot images and densitometric analysis of F-ORF28 in 293T cells cotransfected with the empty vector or pCMV-F-ORF16. Relative band intensities of Western blots for F-ORF28 were measured and shown in the right panel (Actin used as a normalizing protein). Data are presented as means ± SD. * $p < 0.05$ (Mann-Whitney U test; $n = 3$). (B) Quantitative RT-PCR analysis of F-ORF28 mRNA transcripts in 293T cells cotransfected with the empty vector or pCMV-F-ORF16. The threshold cycle (Ct) values of PCR amplification of F-ORF28 and 18S rRNA (as an internal reference gene) for each sample in three independent experiments are shown in the left panel. RT(–) and RT (+) indicate samples without and with the addition of reverse transcriptase, respectively. Detailed procedures regarding quantitative RT-PCR are described in the Supplementary Methods and Materials (see **2.1.** Quantitative reverse transcription-PCR). Data are presented as means ± SD. ns = not signifcant (Mann-Whitney U test; $n = 3$). (C) Representative Western blot images and densitometric analysis of

F-ORF28 in 293T cells cotransfected with pCMV-GFP or pCMV-GFP-ORF16. Relative band intensities of Western blots for F-ORF28 were measured and shown in the right panel (Actin used as a normalizing protein). Data are presented as means ± SD. * $p < 0.05$ (Mann-Whitney *U* test; $n = 3$). (D) Quantitative RT-PCR analysis of F-ORF28 mRNA transcripts in 293T cells cotransfected with pCMV-GFP or pCMV-GFP-ORF16. The threshold cycle (Ct) values of PCR amplification of F-ORF28 and 18S rRNA (as an internal reference gene) for each sample in three independent experiments are shown in the left panel. RT(–) and RT (+) indicate samples without and with the addition of reverse transcriptase, respectively. Data are presented as means ± SD. ns = not signifcant (Mann-Whitney *U* test; $n = 3$). (E) Effects of F-ORF16 on the protein abundance of F-ORF28, F-ORF6, F-ORF52 and F-ORF55 in 293T cells. Notably, Western blot analysis revealed that F-ORF16 specifically increases the protein abundance of F-ORF28, but not F-ORF6, F-ORF52 and F-ORF55. The experiments were repeated twice independently with similar results.

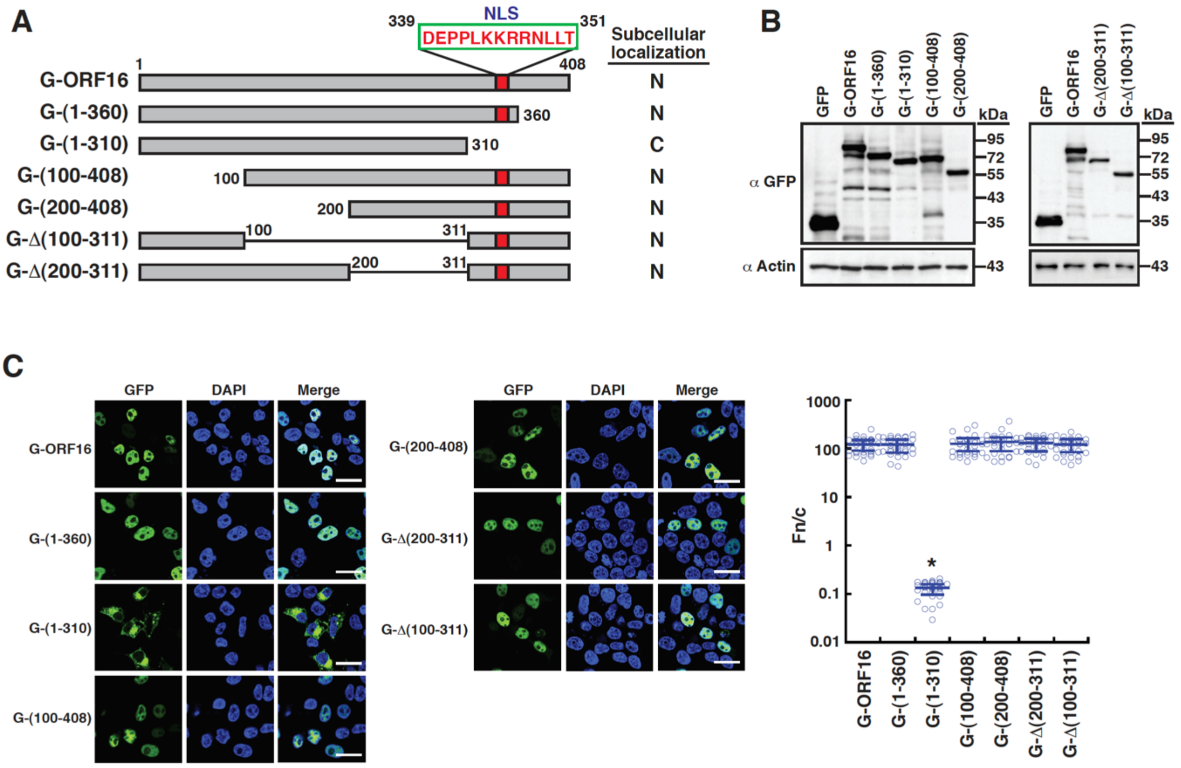

Supplementary Fig. S4. Expression and subcellular localization of wild-type and mutant GFP-ORF16. (A) Schematic diagram of GFP-ORF16 deletion mutants. A predicted NLS is located in the region between aa 339 and 351. The subcellular localization of GFP-ORF16 deletion mutants is summarized in the diagram. (B) Western blot analysis of the expression of GFP-ORF16 deletion mutants expressed in 293T cells. The experiments were repead at least twice independently with similar results. (C) Confocal microscopic analysis of the subellular localization of GFP-ORF16 deletion mutants in 293T cells. Scale bars, 20 μm. The ratios of nuclear to cytoplasmic fluorescence (Fn/c) of GFP-ORF16 and its deletion mutants in individual cells were calculated and shown in the right panel. Data are presented as means ± SD (n = 30 cells, three independent experiments). * $p < 0.0001$ versus the wild-type GFP-ORF16 (two-sample t-test).

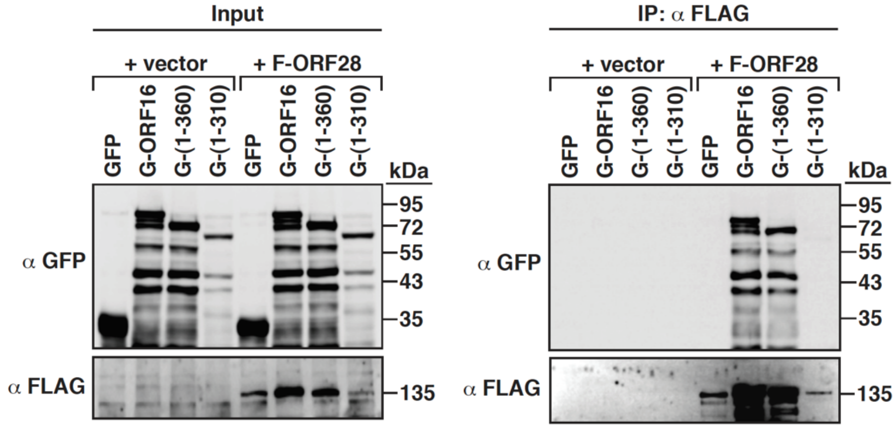

Supplementary Fig. S5. Coimmunoprecipitation analysis of the interaction between F-ORF28 and GFP-ORF16 deletion mutants. In the experiments, 293T cells were cotransfected with different GFP-ORF16 mutants and pCMV-F-ORF28 or the empty vector. At 24 h after transfection, the protein lysates of the transfected cells were prepared and immunoprecipitated using anti-FLAG antibody. Detailed procedures of the coimmunoprecipitation assay are described in Supplementary Methods and Materials (**2.2.** Coimmunoprecipitation with an anti-FLAG antibody). The resultant immunoprecipitates were then analyzed by Western blotting using anti-GFP or anti-FLAG antibody.

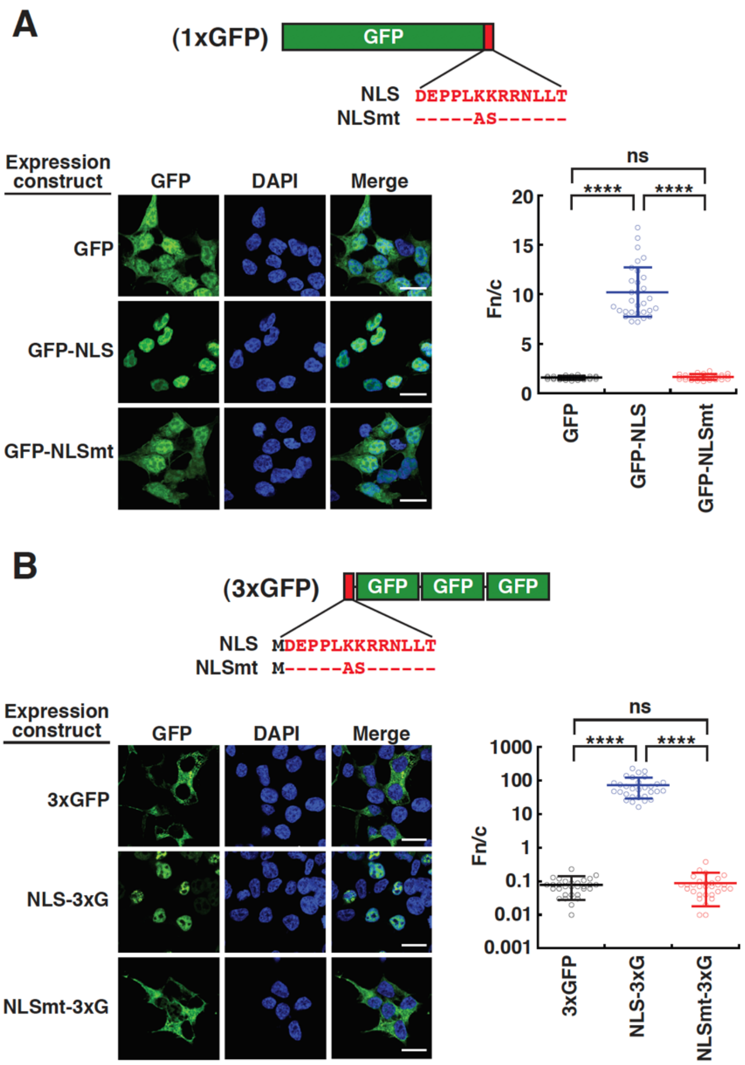

Supplementary Fig. S6. The basic amino acid motif (^{339}DEPPLKKRRNLLT^{351}) of ORF16 is a functional nuclear localization signal. (A) Confocal microscopic analysis of the subellular localization of GFP, GFP-NLS and GFP-NLSmt in 293T cells. Scale bars, 20 μm. The top panel shows the fusion of a 13-amino acid NLS-like motif or a mutated motif to GFP, resulting the generation of GFP-NLS and GFP-NLSmt, respectively. The Fn/c values of GFP, GFP-NLS and GFP-NLSmt in transfected cells were calculated and shown in the right panel. Data are presented as means ± SD (n = 30 cells, three independent experiments). ****$p < 0.0001$, ns = not significant (two-sample t-test). (B) Confocal microscopic analysis of the subellular localization of 3×GFP, NLS-3×GFP and NLSmt-3×GFP in 293T cells. Scale bars, 20 μm. The top panel shows the fusion of a NLS-like motif or a mutated motif to three copies of GFP (3×GFP), thereby generating NLS-3×GFP and NLSmt-3×GFP, respectively. The Fn/c valuses of 3×GFP, NLS-3×GFP and NLSmt-3×GFP in transfected cells were calculated and shown in the right panel. Data are presented as means ± SD (n = 30 cells, three independent experiments). ****p < 0.0001, ns = not significant (two-sample t-test).

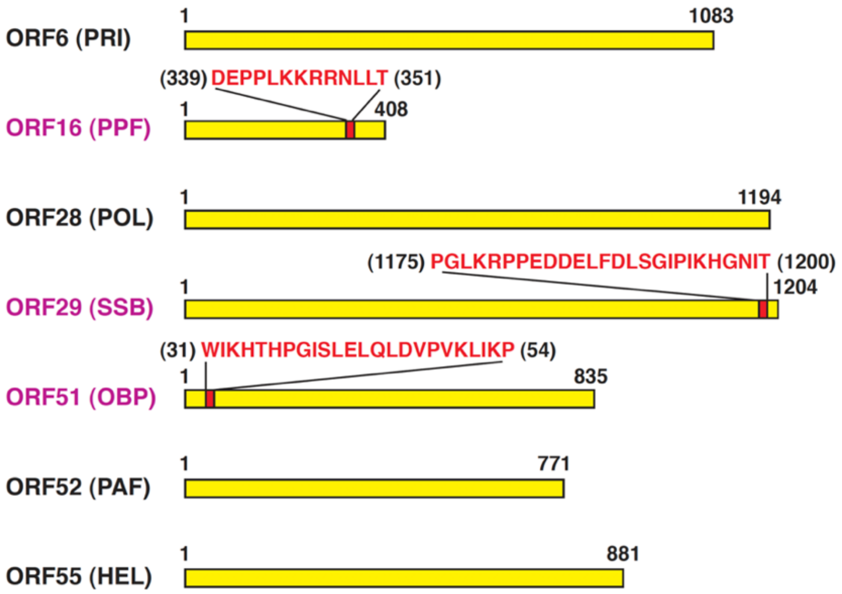

Supplementary Fig. S7. Schematic diagram of VZV replication proteins and the predicted NLSs. The NLS motifs in the VZV replication proteins were predicted using cNLS Mapper (https://nls-mapper.iab.keio.ac.jp/cgi-bin/NLS_Mapper_form.cgi). Detailed information regarding the NLS prediction is mentioned in Supplementary Methods and Materials (**2.3.** NLS prediction using cNLS Mapper program). It should be noted that ORF16 (PPF), ORF29 (SSB), and ORF51 (OBP) contain a predicted NLS motif. The predicted NLS motif (^{339}DEPPLKKRRNLLT^{351}) of ORF16 is a monopartite NLS with a score of 11. However, the predicted NLS motifs of ORF29 (with a score of 5.6) and ORF51 (with a score of 7.4) are bipartite NLSs.

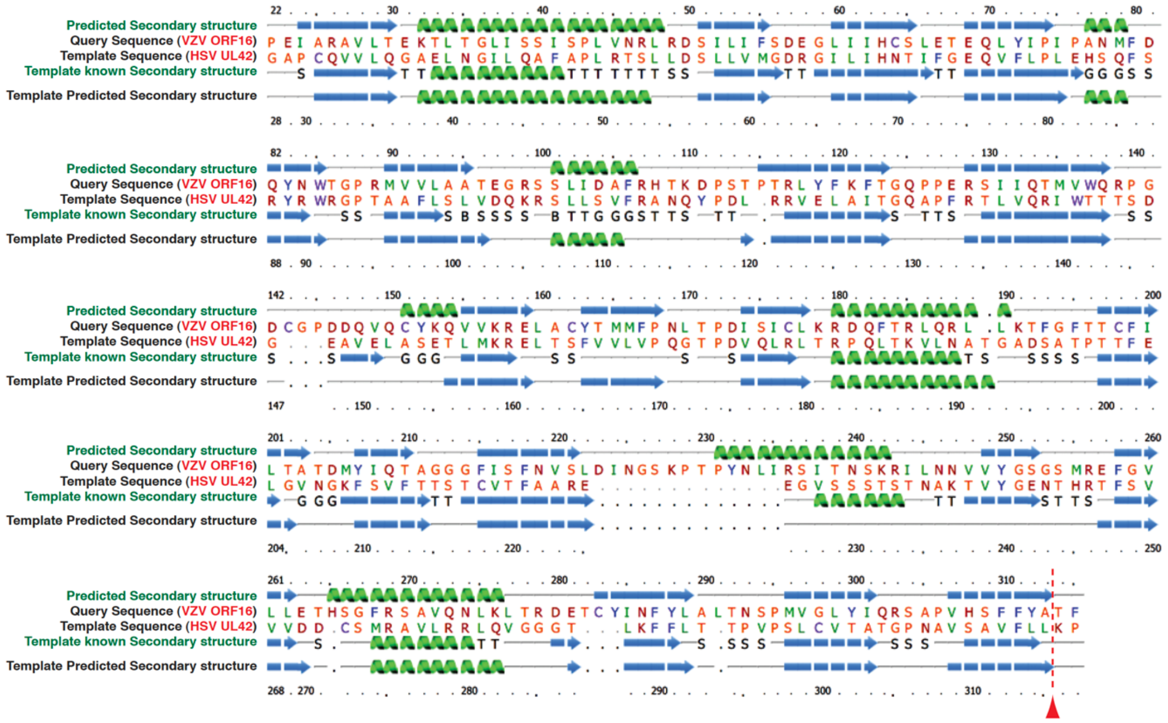

Supplementary Fig. S8. Structural predication of VZV ORF16 using Phyre2 system. The secodary structure prediction using Phyre2 system (http://www.sbg.bio.ic.ac.uk/phyre2/html/page.cgi) shows that the protein region from aa 22 to 315 of VZV ORF16 is structurally similar to the protein region from aa 28 to 317 of HSV-1 UL42. Detailed information regarding the protein structural prediction is descrbed in Supplementary Methods and Materials (**2.4.** Protein stucture prediction using Phyre2 system). As noted, the N-terminal 315 amino acids (marked with an arrowhead) of the HSV-1 UL42 protein is known as a minimal region essential for the interaction with its cognate DNA polymerase UL30.

2. Supplementary Methods and Materials

2.1. Quantitative reverse transcription-PCR (RT-qPCR)

Total RNAs were isolated from transfected cells (2×10^6 cells) using RNeasy Mini Kit (no. 74104; QIAGEN) in combination with the RNase-free DNase set (no. 79254; QIAGEN). An amount of 1 μg of total RNAs from samples was reversely transcribed into cDNA using RevertAid First Strand cDNA Synthesis kit (no. K1622; Thermo Fisher Scientific). The reaction mixtures were prepared in a total volume of 20 μl containing 5 μl of extracted RNA, 5 μM of Random hexamer primer, 200 units of reverse transcriptase, 20 units of RNase inhibitor, 1 mM of dNTP mix, and 1× reaction buffer. The RT reaction mixtures without the addition of reverse transcriptase were used as negative controls. Subsequently, the reverse transcription was performed at 25°C for 5 min for priming, and then at 42°C for 60 min for reverse transcription, and finally the reaction was inactivated at 70°C for 5 min.

Quantitative PCR was performed using iQ SYBR Green Supermix (no. 1708882; Bio-Rad) according to the manufacturer's instructions. The PCR mixtures (25 μl) containing the diluted cDNA template (equivalent to 1 to 25 ng of total RNA), forward and reverse primers (0.8 μM) and 2x iQ SYBR Green Supermix were prepared and subject to PCR amplification using the CFX96 Real-Time PCR Detection System (Bio-Rad). The PCR condition was set including pre-denaturation at 95°C for 1 min, then 35 cycles of denaturation at 95°C for 20 seconds, annealing at 52°C for 30 seconds, and extension at 72°C for 30 seconds. The PCR primers used in the study were following: 5'-CTGCAACACGACCGCTTGGTACCG-3' and 5'-GCCACAAGCGGTATCCACCTCGGC-3' for F-ORF28 (134-bp PCR product); 5'-GGATGTAAAGGATGGAAAATACA-3' and 5'-TCCAGGTCTTCACGGAGCTTGTT-3' for 18S rRNA (72-bp PCR product; GenBank Accession number: NM_022551.3). All qPCR reactions were performed in duplicate. The efficiencies of real-time PCR for the 18S rRNA amplification were from 97 to101%, whereas the efficiencies of real-time PCR for ORF28 amplification were from 96 to102% (representative data shown below; 10-fold serial dilution of template DNA). Relative changes of ORF28 mRNA transcripts were quantified by normalizing to 18S rRNA (as a reference gene) from at least three independent experiments. Fold change was calculated by the $2^{-\Delta\Delta Ct}$ method, where $\Delta\Delta Ct = \Delta Ct_{(effector)} - \Delta Ct_{(control)}$ and $\Delta Ct = Ct_{(ORF28)} - Ct_{(18S\ rRNA)}$.

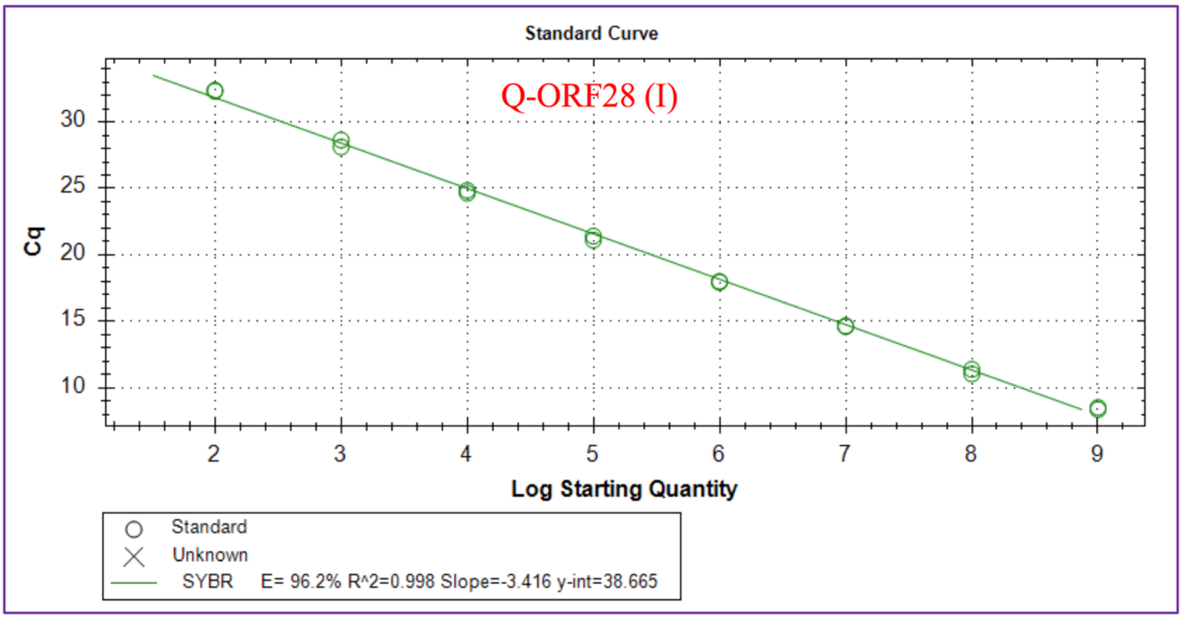

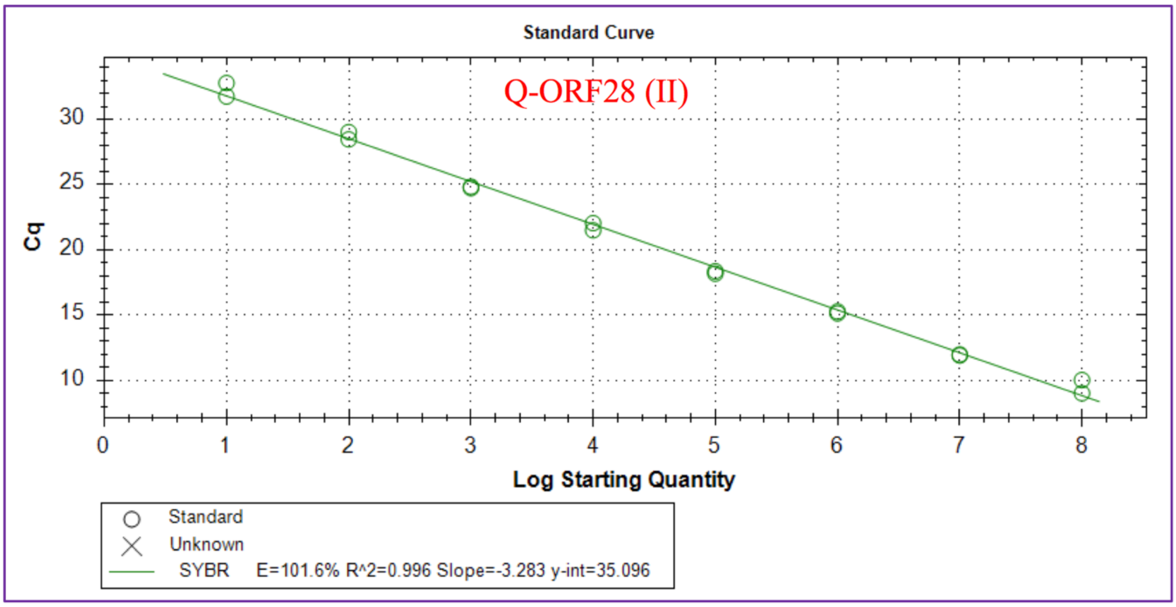

2.2. Coimmunoprecipitation experiments with an anti-FLAG antibody

After transfection of the expression plasmids into 293T cells for 24 h, the transfected cells were subjected to lysis using an immunoprecipitation assay buffer containing 50 mM Tris-HCl (7.5), 150 mM NaCl, 1 mM EDTA, and 1% Triton X-100. Protein extracts were immunoprecipitated using anti-FLAG M2 Magnetic Beads (no. M8823; Sigma-Aldrich), and then the immunoprecipitates were extensively washed using immunoprecipitation washing buffer containing 50 mM Tris-HCl (pH 7.5), 150 mM NaCl, 1 mM EDTA, 0.5% NP-40, and 10% glycerol. The immunoprecipitated proteins were analyzed by Western blotting using anti-FLAG M2-Peroxidase antibody (no. A8592; Sigma-Aldrich) or anti-GFP antibody (no. G1544; Sigma-Aldrich).

2.3. NLS prediction using cNLS Mapper program

The cNLS Mapper program (Kosugi et al., 2009) was used to predict putative NLSs in VZV replication proteins. For predicting NLSs using cNLS Mapper (https://nls-mapper.iab.keio.ac.jp/cgi-bin/NLS_Mapper_form.cgi), the cut-off score of 5.0 was used and the entire region of a protein was selected to search for bipartite NLSs with a long linker region.

2.4. Protein structure prediction using Phyre2 system

To predict the protein structure of VZV ORF16, the Phyre2 system (Kelley et al., 2015) in "Normal" mode was used. The Phyre2 server is available at http://www.sbg.bio.ic.ac.uk/phyre2/html/page.cgi.

3. Supplementary References

Kelley, L.A., Mezulis, S., Yates, C.M., Wass, M.N., Sternberg, M.J., 2015. The Phyre2 web portal for protein modeling, prediction and analysis. Nat Protoc 10(6), 845-858.

Kosugi, S., Hasebe, M., Tomita, M., Yanagawa, H., 2009. Systematic identification of cell cycle-dependent yeast nucleocytoplasmic shuttling proteins by prediction of composite motifs. Proc Natl Acad Sci U S A 106(25), 10171-10176.
